# Significant impact of threshold adjustments on microbiome characterization following Nanopore sequencing

**DOI:** 10.1093/ismeco/ycag219

**Published:** 2026-08-08

**Authors:** Minjie Fu, Yeojoo Yoon, Haeun Cho, Piotr G Jablonski, Sang-im Lee, Jae Chun Choe

**Affiliations:** Interdisciplinary Program of EcoCreative, Ewha Womans University, Seoul 03760, Republic of Korea; School of Biological Sciences, Seoul National University, Seoul 08826, Republic of Korea; Interdisciplinary Program of EcoCreative, Ewha Womans University, Seoul 03760, Republic of Korea; Interdisciplinary Program of EcoCreative, Ewha Womans University, Seoul 03760, Republic of Korea; Museum and Institute of Zoology, Polish Academy of Sciences, Warsaw 00-818, Poland; Department of New Biology, Daegu Gyeongbuk Institute of Science and Technology, Daegu 42988, Republic of Korea; Interdisciplinary Program of EcoCreative, Ewha Womans University, Seoul 03760, Republic of Korea

**Keywords:** long-read sequencing, Oxford Nanopore Technologies (ONT), 16S rRNA gene, real-time microbiome characterization, EPI2ME Agent, filtering thresholds, wild birds, *Parus minor*

## Abstract

Sequencing the full-length 16S rRNA gene is essential for improving the taxonomic resolution of bacterial identification. Long-read Oxford Nanopore Technologies (ONT) sequencing, coupled with its real-time analysis platform EPI2ME, has become increasingly competitive in microbial community profiling studies. However, the impact of threshold settings in EPI2ME Agent on the alignment-based confidence (hereafter “Alignment accuracy”) and the proportion of reads successfully assigned to a taxon (hereafter “Classification success”) has not yet been systematically evaluated. In this study, we performed ONT-based full-length 16S rRNA gene sequencing on samples collected from eggshells of wild Oriental Tits (*Parus minor*). By adjusting different threshold settings in the EPI2ME Agent, we examined how filtering conditions affect Alignment accuracy, Classification success, and downstream microbiome diversity analysis. Our results revealed that filtering thresholds had substantial effects on these outputs. These findings highlight the importance of carefully selecting filtering parameters in the EPI2ME Agent to balance alignment accuracy and classification success, and to improve the interpretability and consistency of taxonomic profiles generated from ONT sequencing data.

## Introduction

Rapid advancements in sequencing technologies have greatly enhanced environmental microbiome research, deepening our understanding of the ecological roles of microbes [1]. When conducting the bacterial community profiling analysis, several factors are often taken into consideration, including sequencing length, speed, price, and accuracy [1]. The reliability of taxonomic and ecological characterization can be influenced by multiple variables, including read accuracy and downstream analytical steps such as quality filtering. Common strategies to improve confidence in taxonomic assignments include rigorous quality control, removal of low-abundance taxa, and increasing sequencing accuracy [2].

Conventional bacterial profiling often relies on next-generation sequencing of partial 16S rRNA gene amplicons (e.g. V3–V4 regions), which provides high read accuracy (~99%) at relatively low cost [1, 3, 4]. However, such short-read amplicon sequencing has limited taxonomic resolution, often restricted to the genus level, and could be affected by primer bias, whereby differential primer–template matching may lead to preferential amplification of specific taxa, thereby distorting downstream species- and strain-level taxonomic profiles [4–7]. To accurately identify microbes at the species or strain level, which is necessary for understanding host–microbe interactions and ecological fitness, at least full-length sequencing of 16S rRNA is required [6, 8].

Long-read sequencing of the full-length 16S rRNA gene using Oxford Nanopore Technologies (ONT) offers rapid, cost-effective, and high-resolution species-level characterization [4, 8]. Although ONT sequencing initially exhibited lower accuracy compared to Illumina platforms [9], substantial improvements in chemistry and basecalling have increased read accuracy from ~60% in 2014 to >90% by 2018 [10]. Recent improvements in ONT sequencing chemistry (e.g. R10.4.1 pores with Q20+ kits) have substantially increased raw read accuracy, with modal accuracies approaching ~99% under optimized conditions. As a result, ONT sequencing has become a competitive and reliable technique for both biomedical and environmental research [7, 8, 11–18].

The EPI2ME “What’s In My Pot” workflow is a cloud-based analysis platform developed by ONT that provides real-time species-level identification from ONT sequencing data through an intuitive graphical interface. Its accessibility and minimal bioinformatics requirements have driven widespread adoption among ONT users (source: https://nanoporetech.com/resource-centre/epi2me-wimp-workflow-quantitative-real-time-species-identification-metagenomic). Compared to Illumina-based workflows, ONT sequencing coupled to EPI2ME has demonstrated consistent performance in identifying dominant operational taxonomic units within the gut microbial community [19] and superior resolution for specific taxa such as *Bifidobacterium* [8].

Despite these advantages, many studies rely on default or partially adjusted EPI2ME Agent filtering settings without systematically evaluating their effects on downstream microbiome inference [15, 20–25]. This represents a critical gap, as parameter selection may influence not only taxonomic assignment but also ecological interpretation.

The EPI2ME Agent allowed users to adjust several key filtering parameters: (i) the *Min qscore* (minimum quality score), default at 7, defines the threshold below which reads fail quality control (https://labs.epi2me.io/quality-scores/); (ii) *Min coverage*, default at 30%, defines the minimal sequence coverage required for a valid match; (iii) *Min identity*, default at 77%, defines the minimal BLAST alignment identity required for a match. Given the broad ranges available for these parameters, it is important to understand how parameter variation influences alignment-based confidence and the stability of taxonomic outputs, rather than assuming default settings are optimal.

Here, we used ONT sequencing of the full-length 16S rRNA gene amplicons from eggshell surface samples of the Oriental Tit (*P. minor*) to characterize microbial communities. Bacterial communities residing on the surface of eggshells can impact egg hatching rates and the fitness of nestlings [26]. In this study, we focused on how analytical parameter choices affected alignment-based confidence and the consistency of microbiome inferences within a widely used analysis framework, rather than attempting to benchmark absolute taxonomic alignment accuracy against a known ground-truth reference. Accordingly, this study was framed as a sensitivity and stability analysis of taxonomic outputs under parameter variation within the EPI2ME Agent pipeline. Specifically, we systematically examined how varying EPI2ME filtering thresholds influenced (i) Alignment accuracy (EPI2ME terminology, defined as sequence similarity to reference databases rather than empirically validated correctness) and Classification success (defined as the proportion of reads successfully assigned to a taxon), (ii) microbiome diversity, and (iii) taxonomic detection across different abundance cutoffs.

## Materials and methods

### Sample collection

Eggshell swabs (*N* = 41) were collected from Oriental Tits (*P. minor*) at the National Institute of Forest Science, Seoul, Republic of Korea, in 2020. Samples were collected in the early morning between 7 and 11 a.m. Swabs were obtained from intact eggshells using sterile cotton swabs following a previously described protocol [27]. Negative control swabs (swab blanks) were collected and processed alongside samples to monitor potential environmental and handling contamination. Samples were immediately placed on ice, transported to the laboratory, and stored at −20°C until DNA extraction. All procedures were approved by the Institutional Animal Care and Use Committee of Seoul National University (No. SNU-180727-1).

### DNA extraction

Genomic DNA was extracted from these eggshell swabs using the FastDNA Spin kit for Soil (MP Biomedicals, Santa Ana, CA, USA) following the manufacturer’s protocol with slight adjustments [28]. In addition to swab blanks, extraction blanks were included during DNA extraction as negative controls. DNA concentration and purity were initially assessed using the NanoDrop 2000 spectrophotometer (Thermo Fisher Scientific, Waltham, MA, USA) and subsequently quantified using a Qubit® 2.0 Fluorometer (Life Technologies, Carlsbad, CA, USA) with Qubit® dsDNA HS assay kits (Molecular Probes, Invitrogen, Eugene, OR, USA).

### 16S rRNA gene amplification and library construction

The full-length 16S rRNA gene (V1–V9 region) was amplified using the 16S Barcoding kit (SQK-16S024; Oxford Nanopore Technologies—ONT, Oxford, UK). PCR amplification was performed using LongAmp® Taq 2× master mix (New England Biolabs, Ipswich, MA, USA) on a Bio-Rad T100™ Thermal Cycler (Bio-Rad Laboratories, Hercules, CA, USA). PCR conditions included an initial denaturation step at 95°C for 5 min, followed by 40 cycles of denaturation at 95°C for 20 s, annealing at 55°C for 30 s, and extension at 65°C for 2 min, with a final extension at 65°C for 15 min. Both swab blanks and extraction blanks failed PCR amplification and therefore were excluded from library construction.

PCR products were verified by electrophoresis, purified using ExoSAP-IT™ PCR Product Cleanup Reagent (Applied Biosystems™; Thermo Fisher Scientific, USA), and further cleaned with AMPure® XP (Beckman Coulter, Brea, CA, USA). Purified amplicons were quantified using Qubit® dsDNA HS assay kits (Molecular Probes, Invitrogen, Eugene, OR, USA) in Qubit® 2.0 Fluorometer (Life Technologies, Carlsbad, California, USA). Barcoded samples were pooled into two libraries (Library 1, *N* = 17; Library 2, *N* = 24). Each library contained up to 96 fmol of amplicons.

### Nanopore sequencing and basecalling

The adapter-ligated libraries were loaded onto SpotON flow cells (FLO-MIN106D, R9.4.1; Oxford Nanopore Technologies—ONT, Oxford, UK) and sequenced for 12 h using MinKNOW (MinION software, ver. 21.06.0) on a MinION™ Mk1B device (Oxford Nanopore Technologies—ONT, Oxford, UK). Basecalling was performed in real-time via Guppy (ver. 5.0.11) integrated within MinKNOW. Reads with quality scores <7 (Library 1) and 8 (Library 2) were filtered out.

### Sequencing analysis: parameters and variables

FASTQ files generated after basecalling were analyzed through the EPI2ME Agent platform (ver. 3-3.4.0-5 818 340; Oxford Nanopore Technologies—ONT, Oxford, UK) against the NCBI RefSeq database (Dec. 2021). Reads ranging from 1400 to 1700 bp (covering V1–V9 regions of the 16S rRNA gene; ~1500 bp) were retained. The BLAST E-value threshold was set to the default [e = 0.01], and the maximum number of target sequences was set to 3 (default).

To investigate the impact of different filtering parameters on the sequencing analysis outcomes, we systematically varied three key parameters: *Min qscore, Min coverage*, and *Min identity*. Specifically, *Min qscore* values of 7 (where applicable), 8, and 9; *Min coverage* values of 30%, 60%, and 80%; and *Min identity* values of 77%, 90%, and 95% were tested. These combinations resulted in 11 treatment conditions (T1 to T11), in addition to the default condition (T0) (Table S1), for each library. For each condition, values for sequencing reads, Alignment accuracy, Classification success, and the number of phyla were obtained from the EPI2ME Agent output. The selected parameter combinations were designed to represent progressively increasing levels of filtering stringency relative to the default EPI2ME settings. After evaluating all tested conditions, representative conditions (T0, T1, and T2) were selected to evaluate the practical effects of threshold adjustment on microbiome profiling outcomes within computationally feasible limits. T0, T1, and T2 (Table 1) were emphasized because they represented the three dominant response patterns observed across the tested conditions, corresponding broadly to default, moderate, and stringent filtering states.

**Table 1:** Nanopore sequencing analysis comparison in the EPI2ME Agent by adjusting different parameters.

	Group ID	*Min_qscore*	*Min_coverage*	*Min_identity*	Alignment accuracy	Classification success	Average quality	Workflow success	No. of phyla
Library 1	T0	7	30	77	86.14	99	10.37	65.3	23
	T1	9	60	90	92	41	10.22	47.1	13
	T2	9	60	95	96	1	10.22	47.1	6
Library 2	T0	7	30	77	85.41	99	10.59	87.5	24
	T1	9	60	90	92	61	10.6	76.5	12
	T2	9	60	95	95	3	10.6	76.7	8

*Min qscore, Min coverage*, and *Min identity* were the filtering parameters varied among treatments. Analyses were based on 17 samples per treatment for Library 1 (51 observations across T0–T2) and 24 samples per treatment for Library 2 (72 observations across T0–T2). Library 2 reads were basecalled and pre-filtered at Q ≥8 prior to EPI2ME analysis.

### Statistical analysis for Alignment accuracy and Classification success

To assess the effects of filtering thresholds on taxonomic assignment, we evaluated alignment-based confidence (hereafter Alignment accuracy) and Classification success. Alignment accuracy was defined as the alignment-based score reported by the EPI2ME pipeline, reflecting sequence similarity to reference database entries and not representing empirically validated taxonomic correctness. Classification success was defined as the proportion of reads successfully assigned to a taxon. Both Alignment accuracy and Classification success metrics were obtained from the EPI2ME built-in pipeline. Library 1 comprised 17 biological samples and Library 2 comprised 24 biological samples. Each sample was analyzed under multiple EPI2ME filtering conditions, resulting in repeated measurements across treatments.

First, Kruskal–Wallis rank sum tests as exploratory comparisons among filtering conditions and Dunn’s tests of Multiple Comparisons Using Rank Sums (hereafter Dunn’s test) [29] from package “FSA” [30] were performed to compare the Alignment accuracy and Classification success outcomes under different filtering conditions. Additionally, Wilcoxon signed-rank tests were applied for pairwise comparisons between specific treatment groups, and linear regression models from package “MASS” [31] were used to assess the relationship between filtering parameters and sequencing outcomes. To further evaluate the effects of filtering parameters across all observations, generalized linear mixed-effects models (GLMMs) implemented in the package “lme4” [32] were used with filtering parameters as fixed effects and sample ID as a random effect. The analyses were performed as follows.

To test the effect of *Min qscore* (7, 8, 9) on Alignment accuracy and Classification success, we compared the T0, T10, and T11 conditions (Table S1) with a fixed *Min coverage* at 30% and *Min identity* at 77%. Kruskal–Wallis rank sum tests and Dunn’s tests adjusted with the Bonferroni method as above were performed for this statistical analysis.

To test the effect of *Min coverage* on Alignment accuracy and Classification success, we evaluated two comparison groups. The first comparison (Group 1) was between T1 (*Min coverage*, 60%) and T3 (*Min coverage*, 80%), with *Min qscore* fixed at 9 and *Min identity* fixed at 90% (Table S1). The second comparison (Group 2) was between T5 (*Min coverage*, 80%) and T8 (*Min coverage*, 30%), with *Min qscore* fixed at 8 and *Min identity* fixed at 95%. Wilcoxon signed-rank tests with continuity correction were used for both comparisons.

To test the effect of *Min identity* on Alignment accuracy and Classification success, we evaluated two comparison groups. The first comparison (Group 1) was between T1 (*Min identity*, 90%) and T2 (*Min identity*, 95%), with *Min qscore* fixed at 9 and *Min coverage* fixed at 60% (Table S1). The second comparison (Group 2) was between T0 (*Min identity*, 77%) and T7 (*Min identity*, 95%), with *Min qscore* fixed at 7 and *Min coverage* fixed at 30%. Wilcoxon signed-rank tests with continuity correction were used for both comparisons.

### Assessing phyla detection

To investigate the effects of the filtering parameters on the detection of phyla, the number of phyla detected in each sample was first recorded at various abundance cutoffs (0%, 0.1%, 0.5%, 1%, 3%) using the EPI2ME Agent. Then, GLMMs were applied to evaluate the effects of *Min qscore, Min coverage*, and *Min identity* on phylum detection under different cutoff conditions.

### Statistical analysis for microbiome diversity and structure

Normalized alpha diversity metrics calculated from relative abundance data, including richness, Shannon’s diversity index, Simpson’s index, and Pielou’s evenness, as well as beta diversity based on Bray–Curtis distances, were assessed for T0, T1, and T2 conditions (Table 1) at the phylum, genus, and species levels under abundance cutoffs of 0.5%, 1%, and 3%.

Kruskal–Wallis rank sum tests followed by Dunn’s tests with Bonferroni correction [29] were used to compare alpha diversity and taxon abundances. Beta diversity differences were assessed using permutational multivariate analysis of variance (PERMANOVA) on Bray–Curtis dissimilarities with 9999 permutations after accounting for repeated measurements using restricted permutations by sample ID [33]. Non-metric multidimensional scaling (NMDS) [34] was used for displaying the beta diversity among different groups. Detailed analysis results can be found in supplementary results.

All analyses were conducted in R ver. 4.4.1 (R Foundation for Statistical Computing, Vienna, Austria).

## Results

A total of 17 172–492 750 (111 223.3 ± 105 114.2) and 77 449–260 652 (125 862.2 ± 42 523.28) reads were obtained for each sample in Library 1 and Library 2, respectively.

### Alignment accuracy and classification success under different filtering conditions

**Figure 1:**
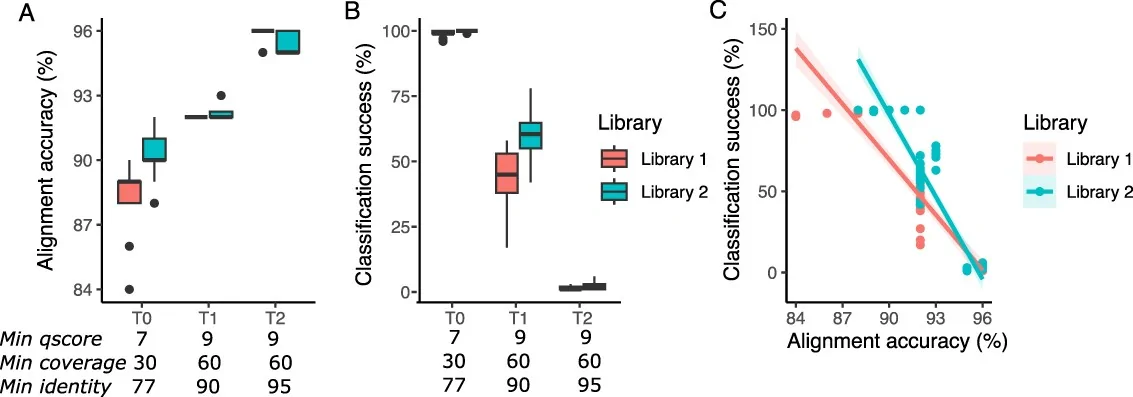
Alignment accuracy and Classification success under different conditions (T0–T2) and their negative relationship. (A) Alignment accuracy under different filtering conditions. (B) Classification success under different conditions. (C) Linear regression plot showing the negative correlation between Alignment accuracy and Classification success. T0 represented the default condition in EPI2ME Agent. T1 and T2 represented increasingly stringent filtering conditions obtained by increasing *Min qscore, Min coverage*, and *Min identity* thresholds. Regression statistics: _adjusted_*R*^2^ = 0.87, *P* < .001; library 1, *β* = −11.38, *F*_1,49_ = 329.40; library 2, *β* = −16.92, *F*_1,70_ = 479.50. Analyses were based on 17 samples per treatment for Library 1 and 24 samples per treatment for Library 2.

Across 12 filtering conditions, both alignment-based confidence scores (Alignment accuracy) and the proportion of reads successfully assigned to a taxon (Classification success) differed significantly in Libraries 1 and 2 (Kruskal–Wallis, *P* < .0001) (Table S1, Fig. S1A–B). Patterns were consistent between libraries (Wilcoxon, *P* > .05) (Fig. S1A–B). Three representative settings (T0–T2) captured the major trends (Table 1, Fig. 1A–B, and  Fig.  S1A–B): (i) T0, the default setting, classified nearly all sequences (>99%) but had the lowest alignment-based confidence (~86%); (ii) T1 yielded moderate Alignment accuracy (~90%) and Classification success (about 50%); (iii) T2 generated the highest alignment-based confidence (~95%) but lowest Classification success (~1%). Additionally, alignment-based confidence and Classification success were negatively correlated in both libraries (_adjusted_*R*^2^ > 0.85, *P* < .001) (Fig. 1C and Fig.  S2, Table S2).

### Effect of individual parameters

**Figure 2:**
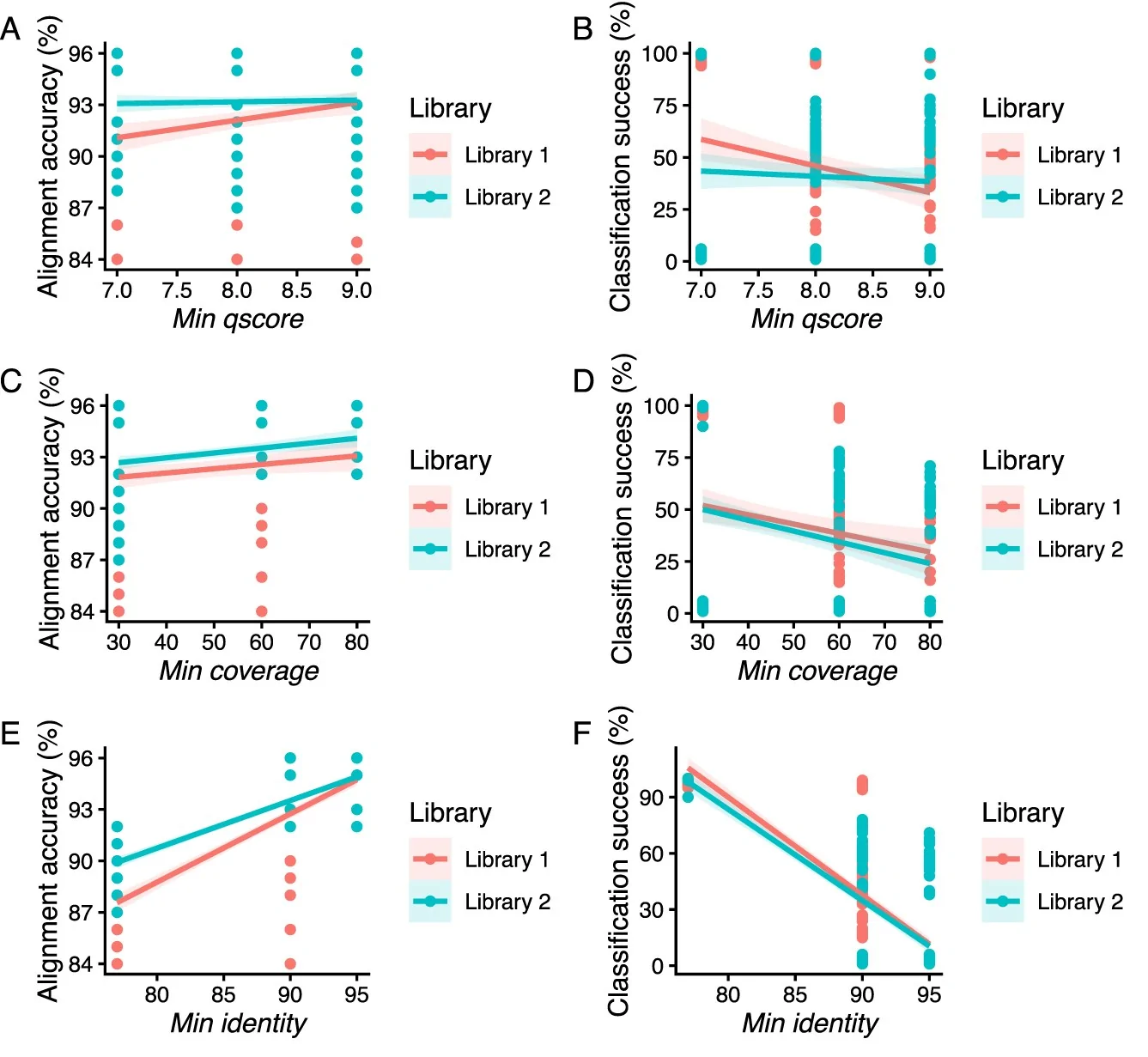
Effects of different parameters on Alignment accuracy and Classification success. Linear regression plots showing the effects of *Min qscore* on Alignment accuracy (A) and Classification success (B), and the effects of *Min coverage* on Alignment accuracy (C) and Classification success (D), as well as the effects of *Min identity* on Alignment accuracy (E) and Classification success (F). These plots were based on data from all 12 filtering conditions (T0–T11). Summary of statistics for linear regression models is provided in Table S2. Analyses were based on 17 samples per treatment for Library 1 (204 observations across T0–T11) and 24 samples per treatment for Library 2 (288 observations across T0–T11).

Linear regression indicated that *Min identity* and *Min coverage* were positively associated with alignment-based confidence and negatively associated with Classification success (*P* < .05), with *Min identity* showing the strongest effect (_adjusted_*R*^2^ > 0.6, *P* < .001) (Fig. 2, Table S2).

GLMM results confirmed that *Min identity* and *Min coverage* significantly influenced both metrics (*P* < .01), whereas *Min qscore* showed minor or inconsistent effects (Tables S3–S7).

### Alpha diversity under different filtering conditions

Phylum detection differed significantly under most abundance cutoffs (Kruskal–Wallis, *P* < .05), except at the 3% cutoff in Library 2 (Fig. S1C–F, Table S8). The effects of individual filtering parameters differed between libraries. Specifically, *Min qscore* influenced phylum detection only in Library 2 across all cutoffs (0%–3%) (Tables S4 and S5), whereas *Min coverage* had a significant effect only in Library 1 (Table S6). In contrast, *Min identity* showed the most consistent influence, significantly affecting the detection of phyla across all cutoffs (0%–3%) in Library 1 (Fig. S1C–F, Table S7) and at lower cutoffs (0%–0.5%) in Library 2 (Fig. S1C–F, Table S7).

**Figure 3:**
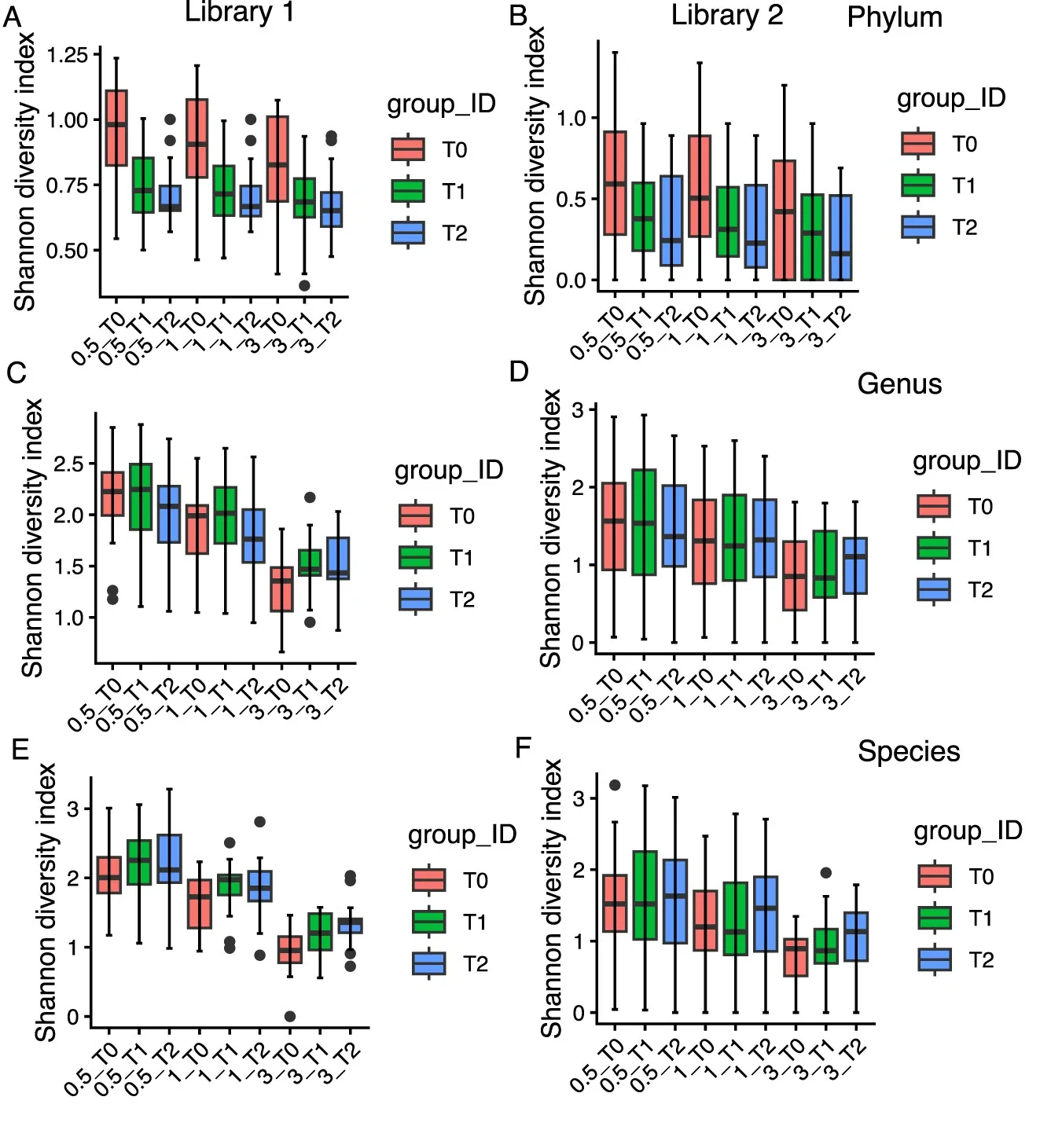
Comparison of alpha diversity (Shannon’s diversity index) under different filtering conditions (T0–T2) and abundance cutoffs in Libraries 1 and 2. Alpha diversity at the phylum level under different cutoffs for Library 1 (A) and Library 2 (B). Alpha diversity at the genus level under different cutoffs for Library 1 (C) and Library 2 (D). Alpha diversity at the species level under different cutoffs for Library 1 (E) and Library 2 (F). 0.5_T0, 0.5% cutoff using T0. 0.5_T1, 0.5% cutoff using T1. 0.5_T2, 0.5% cutoff using T2. 1_T0, 1% cutoff using T0. 1_T1, 1% cutoff using T1. 1_T2, 1% cutoff using T2. 3_T0, 3% cutoff using T0. 3_T1, 3% cutoff using T1. 3_T2, 3% cutoff using T2. Analyses were based on 17 samples per treatment for Library 1 and 24 samples per treatment for Library 2.

For alpha diversity metrics, richness and Shannon diversity differed primarily between the default condition (T0) and the higher-stringency conditions (T1 and T2), particularly at the phylum and species levels across multiple cutoffs (Fig. 3, Table S9). In contrast, differences between T1 and T2 were generally not significant, and genus-level effects were minimal (Fig. 3, Table S9).

Metrics reflecting evenness, including Simpson’s index and Pielou’s evenness, showed no significant differences across filtering conditions in either library (*P* > .05), with the exception of a significant difference between T0 and T2 in Simpson’s index at the species level using the 3% cutoff for Library 1 (Table S9).

### Beta diversity under filtering conditions

**Figure 4:**
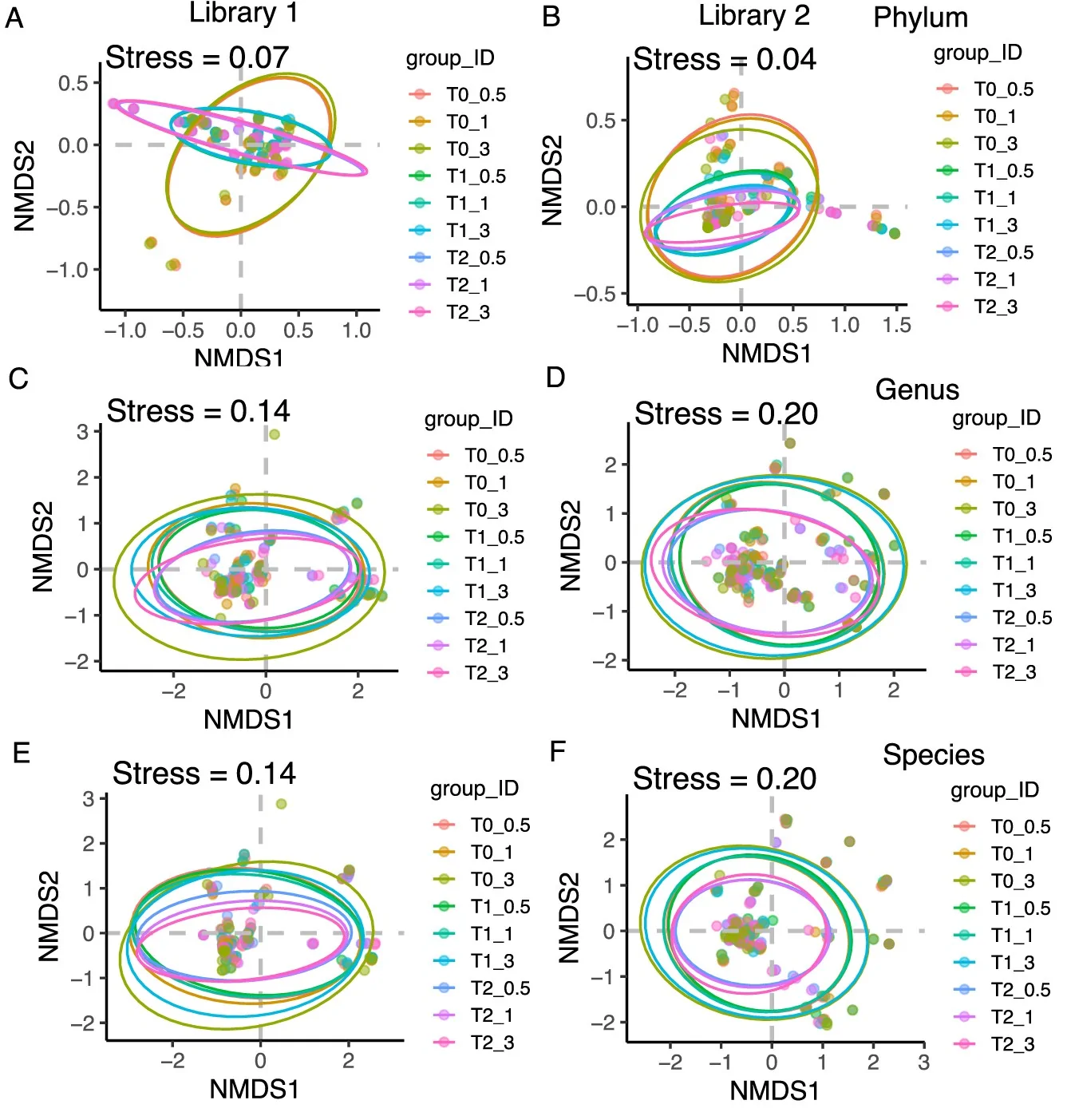
Non-metric multidimensional scaling (NMDS) plots showing microbiome community structure using different filtering conditions (T0–T2) and abundance cutoffs in Libraries 1 and 2. Microbiome community structure at the phylum level for Library 1 (A) and Library 2 (B). Microbiome community structure at the genus level for Library 1 (C) and Library 2 (D). Microbiome community structure at the species level for Library 1 (E) and Library 2 (F). T0_0.5, 0.5% cutoff using T0. T1_0.5, 0.5% cutoff using T1. T2_0.5, 0.5% cutoff using T2. T0_1, 1% cutoff using T0. T1_1, 1% cutoff using T1. T2_1, 1% cutoff using T2. T0_3, 3% cutoff using T0. T1_3, 3% cutoff using T1. T2_3, 3% cutoff using T2. Ellipses indicate 95% confidence intervals. Analyses were based on 17 samples per treatment for Library 1 and 24 samples per treatment for Library 2.

No significant differences in microbial community structure were detected among filtering conditions (T0, T1, and T2) at the phylum, genus, or species levels in either library across all abundance cutoffs (PERMANOVA, *P* > .05) (Fig. 4, Table S10).

### Sensitivity of taxonomic profiles to filtering thresholds and abundance cutoffs

**Figure 5:**
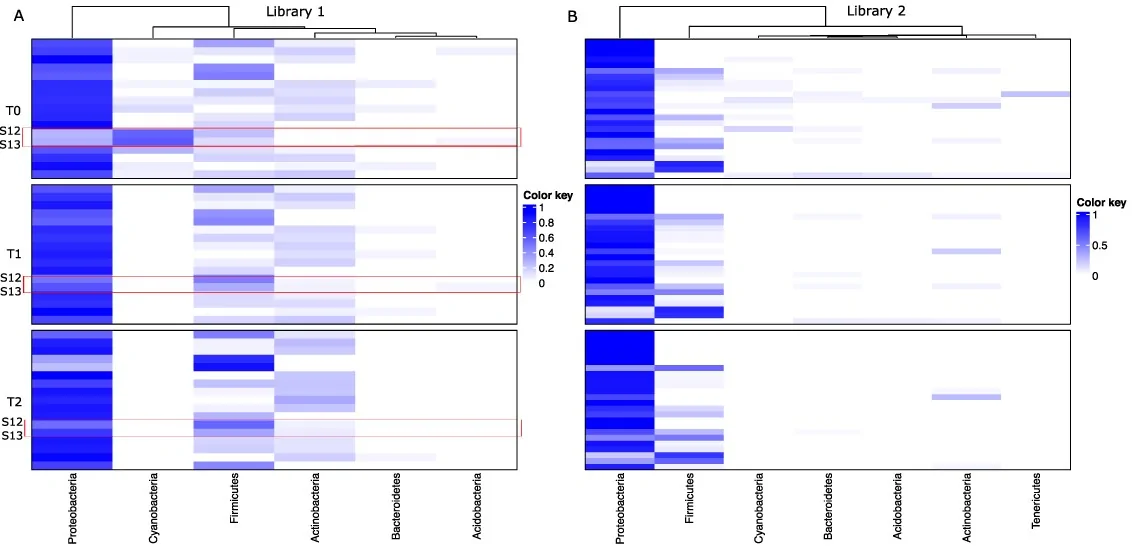
Relative abundance of bacterial phyla under a 3% abundance cutoff in Library 1 (A) and Library 2 (B). Sample IDs were S1–S17 and S1–S24 from top to bottom for each filtering condition in Libraries 1 and 2, respectively. Samples highlighted with a frame were selected for subsequent comparisons of Alignment accuracy among T0, T1, and T2. Analyses were based on 17 samples per treatment for Library 1 and 24 samples per treatment for Library 2.

**Figure 6:**
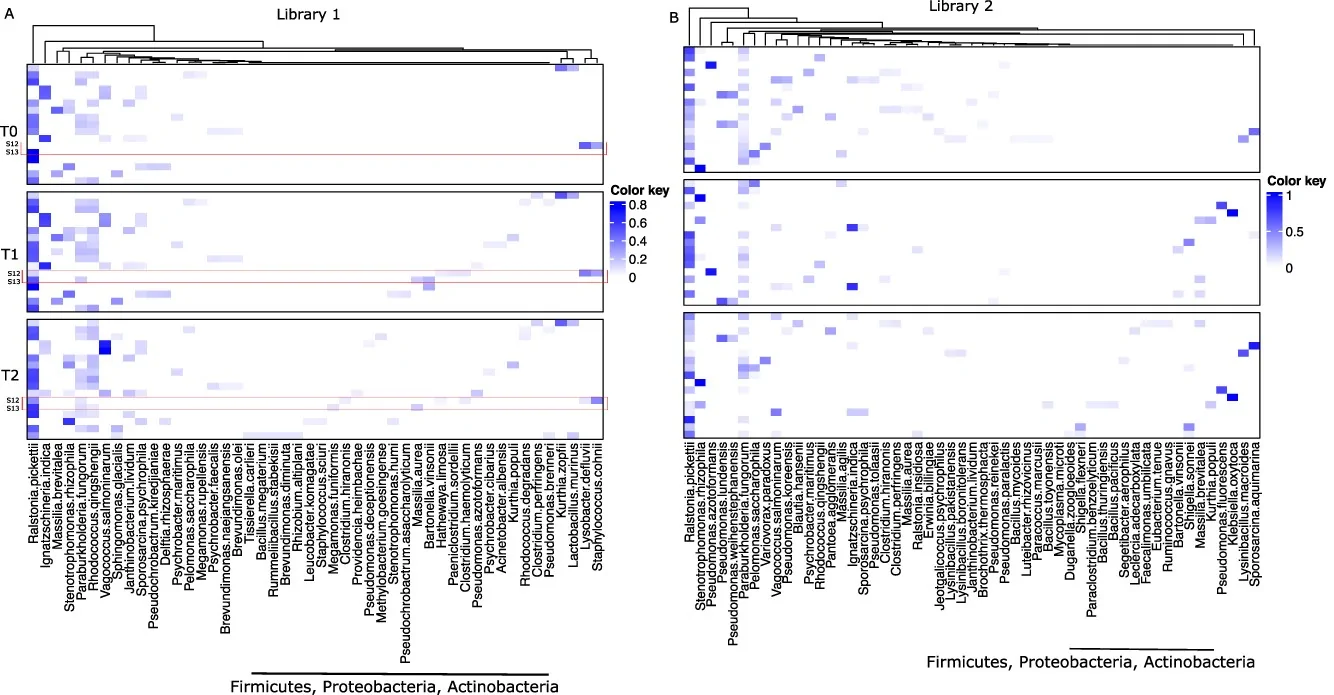
The relative abundance of bacterial species under 3% abundance cutoff in Library 1 (A) and Library 2 (B). Sample IDs were S1–S17 and S1–S24 from top to bottom for each filtering method in Libraries 1 and 2, respectively. Samples highlighted with a frame were selected for subsequent comparisons of Alignment accuracy among T0, T1, and T2. Analyses were based on 17 samples per treatment for Library 1 and 24 samples per treatment for Library 2.

To further assess how filtering thresholds influence taxonomic profiles, we focused on the 3% abundance cutoff in both libraries. Among the top six phyla examined, only Cyanobacteria showed a significant difference (χ^2^ = 24.01, *d.f.* = 2, *P* < .001) across filtering conditions (T0, T1, and T2; Table 1), whereas other dominant phyla (Proteobacteria, Firmicutes, and Actinobacteria) remained stable across different filtering conditions in both libraries (Fig. 5, Table S11). Under the default setting (T0), Cyanobacteria represented the fourth most abundant phylum and was detected in 10 samples out of 17 (58.82%) (Fig. 5). In contrast, Cyanobacteria was completely absent under the higher-stringency conditions (T1 and T2) (Fig. 5). Notably, applying stricter filtering (T1 and T2) was associated with the detection of additional species within dominant phyla (Fig. 6). This pattern corresponded with increases in richness and Shannon diversity observed under T1 and T2 (Fig. 3E, Table S9).

To further investigate this pattern, we examined two samples (S12 and S13) from Library 1. Cyanobacteria assignments showed substantially lower alignment-based confidence compared to Proteobacteria (Fig. S3). Despite high phylum-level relative abundance (Fig. 5A), no species-level assignments were detected in Cyanobacteria for these samples under T0 (Fig. 6A), indicating inconsistency across taxonomic levels under default setting. A detailed analysis of Sample S12 revealed that the dominant Cyanobacteria-related assignments corresponded to the order *Oscillatoriales* (2409 reads) and species *Tychonema bourrellyi* (153 reads), respectively (Table S12). However, *T. bourrellyi* was only detected at low-abundance cutoffs (0% and 0.1%) and was not retained at higher thresholds (≥0.5%) (Fig. S4).

## Discussion

Long-read ONT sequencing has historically been criticized for lower per-read accuracy relative to short-read Illumina platforms [9]. However, continuous improvements in sequencing chemistry and basecalling algorithms have substantially increased its reliability, making it an increasingly competitive approach for complex microbiome analyses [8, 11–13]. In parallel, downstream quality control and filtering strategies are recognized as critical determinants of taxonomic assignment confidence and stability [2].

In this study, we systematically evaluated how key filtering parameters (*Min identity, Min qscore*, and *Min coverage*) in the EPI2ME Agent influenced Alignment accuracy, Classification success, and ecological inference using full-length 16S rRNA Nanopore data. Our analysis focused on relative changes in alignment confidence and consistency across parameter settings rather than on empirically validated taxonomic correctness. The results show that parameter selection has a strong and structured effect on microbiome characterization outcomes. To our knowledge, this is the first study to explicitly compare multiple filtering regimes within the EPI2ME Agent framework for real-time microbiome analysis.

### Balancing Alignment accuracy and Classification success in EPI2ME agent

A central finding of this study is the pronounced trade-off between Alignment accuracy and Classification success (Fig. 1). Increasing stringency improved Alignment accuracy (from 86.1% to 96%) but markedly reduced Classification success from almost 100% to 2% (Fig. 1). It is notable that extremely stringent conditions (e.g. T2, *Min identity* = 95%) resulted in near-complete loss of classified reads. This suggests that a substantial fraction of reads may represent sequences with insufficient similarity to reference databases, potentially reflecting sequencing errors, database incompleteness, or uncharacterized taxa. Therefore, maximizing Alignment accuracy alone may not always be optimal, as parameter selection involves a trade-off between confidence and data retention. This inverse relationship highlights an inherent trade-off in sequence-based taxonomic assignment: stricter filtering may reduce false-positive or low-confidence alignments but simultaneously increase the likelihood of discarding reads that cannot be confidently matched to reference databases.

Among the evaluated parameters, *Min identity* had the strongest and most consistent effect on both Alignment accuracy and Classification success (Fig. 2). Increasing *Min identity* substantially improved alignment confidence but sharply reduced Classification success, indicating that sequence similarity thresholds are a primary determinant of assignment stringency in this framework. In contrast, the effects of *Min qscore* and *Min coverage* were comparatively modest and appeared to depend on library-specific characteristics (Fig. 2, Tables S2–S7), which may be due to differences in sample composition and the quality score thresholds used during sequencing (7 for Library 1 and 8 for Library 2).

At the default *Min identity* threshold (77%), we observed taxonomic patterns that differed from those obtained under more stringent conditions (Figs. 5–6). For instance, Cyanobacteria were detected under the default setting but were not consistently recovered under higher-stringency conditions (Fig. 5). Notably, Cyanobacteria have not been reported from the eggshells of hatching farmed chicks [35] and are typically associated with aquatic environments [36, 37]. In addition, Cyanobacteria assignments exhibited lower alignment confidence than the dominant phyla and inconsistent detection across taxonomic levels (Fig. 5 and Fig.  S3), indicating that these assignments were particularly sensitive to filtering thresholds. Accordingly, the taxonomic profiles generated under more stringent conditions (T1 and T2) showed higher Alignment accuracy and greater consistency across filtering thresholds than those obtained under the default setting (T0), suggesting that they may represent more conservative taxonomic profiles. Under more stringent filtering (T1 and T2), the dominant phyla, Proteobacteria, Firmicutes, Actinobacteria, and Bacteroidetes, were consistently detected, aligning with previous reports on the eggshells of the *P. minor* from the same location [27]. This interpretation is also consistent with the largely unchanged beta diversity across filtering conditions (Fig. 4 and Table S10), suggesting that threshold adjustments primarily affected filtering-sensitive or low-abundance assignments rather than the overall community structure. This concordance suggests that moderate-to-high-stringency settings yield higher alignment confidence and more consistent taxonomic profiles when using the EPI2ME Agent for full-length 16S rRNA amplicon sequencing. However, this interpretation should be treated with caution because no ground-truth reference was available for validation.

Although not all theoretical parameter combinations were evaluated, the tested conditions consistently produced three major response patterns represented by T0, T1, and T2, corresponding to default, moderate, and stringent filtering conditions. These representative groups captured the dominant trends in Classification success and Alignment accuracy across the tested parameter space. Future studies using full-factorial experimental designs may further resolve higher-order interaction effects among filtering parameters.

### Taxa detection, abundance cutoffs, and microbiome diversity

In addition to filtering parameters, abundance cutoffs played a significant role in shaping taxonomic and ecological interpretations [3]. Phylum detection and diversity metrics varied across both filtering thresholds and relative abundance cutoffs, indicating that these two factors interact to influence downstream analyses (Fig. 3 and Fig.  S1C–F). At lower abundance cutoffs, more taxa were retained, increasing sensitivity but also introducing greater variability across filtering conditions; in contrast, higher cutoffs (≥1%) reduced sensitivity to filtering parameters and yielded more stable community profiles (Table S7). This suggests that abundance filtering can partially mitigate the effects of parameter variation by excluding low-abundance taxa that are more sensitive to threshold changes.

Alpha diversity analyses revealed that stricter filtering reduced diversity at higher taxonomic levels (e.g. phylum) under intermediate cutoffs (0.5%), but, somewhat counterintuitively, increased diversity at the species level (Fig. 3). This pattern likely reflects the removal of poorly supported higher-level assignments while improving the taxonomic resolution of retained reads (Figs. 5–6, Fig.  S4). These observations indicate that taxonomic profiles derived from EPI2ME Agent are sensitive to both filtering thresholds and abundance cutoffs, particularly for low-abundance taxa. Taxa detected exclusively under less stringent conditions (e.g. T0) tended to show lower alignment support and inconsistent detection across thresholds, suggesting lower confidence in their taxonomic classification. Increasing abundance cutoffs, in combination with stricter filtering parameters, reduced the contribution of such low-confidence assignments and resulted in taxonomic profiles with higher alignment confidence. However, overall community structure remained largely stable across filtering conditions (Fig. 4). This suggests that alignment filtering primarily influences the detection of specific taxa and diversity estimates rather than causing broad shifts in community composition.

Nevertheless, because no mock community or ground-truth reference was included, misclassification rates could not be directly quantified. Accordingly, our interpretation is limited to relative changes in alignment confidence and consistency across filtering conditions, rather than absolute taxonomic correctness. While trends were consistent across independent libraries, external validation using controlled benchmark datasets will be required to confirm these patterns. In addition, taxonomic assignment depends on the reference database used, and classification outcomes can vary across databases such as NCBI RefSeq, SILVA, and RDP [38, 39]. In this study, all analyses were performed using the same RefSeq database; therefore, observed differences are attributable to parameter variation rather than database choice. Nevertheless, database-specific biases cannot be excluded, and future cross-database comparisons will be important to evaluate robustness. Future comparisons across alternative reference databases (e.g. SILVA) [40], including updated database releases, would help to further evaluate the robustness of these findings.

From an applied perspective, the choice of abundance cutoff should be guided by study objectives. Higher cutoffs (>1%) may be appropriate when focusing on dominant community members, whereas lower cutoffs are necessary when detecting rare or opportunistic taxa, albeit with increased uncertainty [3, 41]. In such cases, stricter filtering (e.g. higher *Min identity)* may help mitigate low-confidence assignments. Although the specific workflow evaluated here (EPI2ME Agent v3.4.0, December 2021 RefSeq database, R9.4.1 flow cells, and Kit 12 chemistry) has since been superseded by newer ONT chemistries, basecalling approaches, and workflow versions, the conceptual trade-offs observed between Alignment accuracy and Classification success are likely relevant to similar filtering-based taxonomic assignment workflows. Nevertheless, the magnitude of these effects may differ under newer software versions, databases, and sequencing chemistries and should therefore be evaluated independently.

## Conclusion

Rather than establishing absolute taxonomic Alignment accuracy, this study highlighted how parameter selection systematically altered confidence metrics and downstream interpretations. Among these parameters, *Min identity* was the primary driver of the trade-off between Alignment accuracy and Classification success, while *Min coverage* and *Min qscore* exerted more context-dependent effects. We further showed that abundance cutoffs interacted with filtering thresholds to shape diversity estimates and taxonomic composition, with higher cutoffs improving stability at the cost of sensitivity. Moderate filtering conditions (e.g. T1) provided a practical balance between Alignment accuracy and sequence retention, whereas highly stringent settings (e.g. T2) may be appropriate when prioritizing confidence over completeness. Overall, this work provides a practical framework for evaluating parameter sensitivity and improving transparency and reproducibility in Nanopore-based microbiome analyses.

## Supplementary Material

Supplementary_Final_ycag219

## Data Availability

The sequencing data are available at the NCBI SRA database under BioProject with an accession number PRJNA1227707.
