# Supplementary material for: Significant impact of threshold adjustments on microbiome characterization following Nanopore sequencing
Source: ISME Commun. 2026 Aug 8;6(1):ycag219. doi: 10.1093/ismeco/ycag219 (PMC13533206; doi:10.1093/ismeco/ycag219)
Supplement: Supplementary file 1 [file supplementary_final_ycag219.docx]

**Significant impact of threshold adjustments on microbiome characterization following Nanopore sequencing**

**Running title: Impact of nanopore filtering thresholds**

Minjie Fu^1,2*†^, Yeojoo Yoon^1^, Haeun Cho^1^, Piotr G. Jablonski^3^, Sang-im Lee^4*^, Jae Chun Choe^1^

^1^ Interdisciplinary Program of EcoCreative, Ewha Womans University, Seoul 03760, Republic of Korea

^2^ School of Biological Sciences, Seoul National University, Seoul 08826, Republic of Korea

^3^Museum and Institute of Zoology, Polish Academy of Sciences, Warsaw 00-818, Poland

^4^Department of New Biology, Daegu Gyeongbuk Institute of Science and Technology, Daegu 42988, Republic of Korea

Present address^†^:

College of Medicine, Seoul National University, 103 Daehak-ro, Jongno-gu, Seoul 03080, Republic of Korea

**Corresponding authors:**

Minjie Fu, College of Medicine, Seoul National University, 103 Daehak-ro, Jongno-gu, Seoul 03080, Republic of Korea. Email: [fumj90@snu.ac.kr](mailto:fumj90@snu.ac.kr)

Sang-im Lee, Department of New Biology, Daegu Gyeongbuk Institute of Science and Technology, 333 Techno Jungang-daero, Hyeonpung-eup, Dalseong-gun, Daegu 42988, Republic of Korea. Email: [sangim@dgist.ac.kr](mailto:sangim@dgist.ac.kr)

**Supplemental Results**

**Effect of individual parameters**

Linear regression analysis using data from all 12 filtering conditions revealed that *Min identity* and *Min coverage* could significantly but weakly increase the Alignment accuracy while decreasing Classification success in certain degree (_adjusted_*R^2^* < 0.10, *p* < 0.05), the effects of *Min identity* on Alignment accuracy and Classification success were much more significant (_adjusted_*R^2^* > 0.6, *p* < 0.001) (Fig. 2, Table S2). GLMM models showed that both *Min identity* and *Min coverage* had significant effects on the Alignment accuracy and Classification success for both libraries (*pseudoR^2^* > 0.65, *p* < 0.01) (Table S3). *Min qscore* only had significant effect on the Alignment accuracy and Classification success in Library 1 (*p* < 0.01) but not in Library 2 (*p* > 0.05) (Table S3). To provide a better comparison, more statistical analyses were performed by fixing two other parameters when comparing one parameter.

***Min qscore*:** Both Kruskal-Wallis rank sum test and Dunn’s pairwise tests revealed no significant differences in *Min qscore’* s effect on the Alignment accuracy and Classification success for both libraries (*p* > 0.05) (Table S4 & S5).

***Min coverage***: Wilcoxon signed rank tests showed *Min coverage* did not affect Alignment accuracy in Library 1 (*p* > 0.05), but significantly affected Alignment accuracy in both groups of Library 2 (*p* < 0.00001) (Table S6). Meanwhile, the tests indicated that Classification success was significantly affected by different *Min coverage* in both libraries (*p* < 0.05) (Table S6). These results suggested that the effect of *Min coverage* on Alignment accuracy may depend on different situations, such as the library.

***Min identity***: Significant effects of *Min identity* were observed on both Alignment accuracy and Classification success by *Min identity* in both groups and libraries (*p* < 0.001) (Table S7). These results suggested that altering the *Min identity* threshold was critical for enhancing Alignment accuracy.

**Alpha diversity across different abundance cutoffs and filtering conditions**

At the phylum level, we found significant differences in Shannon’s diversity index (0.5% cutoff) and richness (0.5%, 1% cutoffs) among the three methods (T0, T1 and T2) for both libraries (*p* < 0.05) (Fig. 3A&B, Table S9). In Library 1, significant differences were also detected in Shannon’s diversity index at 1% cutoff and richness at 3% cutoff (Fig. 3A&B, Table S9).

At the genus level, we only detected significant difference in the richness at 3% cutoff of Library 1 (*p* < 0.05), but no significant differences were found for other groups in both libraries (*p* > 0.05) (Fig. 3C&D, Table S9).

At the species level, we observed consistently significant difference in the richness under 3% cutoff in both libraries (*p* < 0.05) (Table S9). We also detected higher Shannon’s diversity index in T1 and T2 than in that in T0 for Library 1 (Fig. 3E), but not for Library 2 (Fig. 3F) (Table S9).

**Beta diversity across different abundance cutoffs under different filtering methods**

At the phylum level, no significant differences in microbiome community composition were detected among filtering conditions (T0, T1, and T2) at the 0.5%, 1%, or 3% abundance cutoffs in either library (PERMANOVA, *p* > 0.05) (Fig. 4A–B, Table S10). In Library 1, pairwise comparisons among filtering conditions showed moderate effect sizes at the 0.5% cutoff (*R²* = 0.03–0.09) and 1% cutoff (*R²* = 0.03–0.08), but none remained significant under the repeated-measures permutation framework (Table S10). Similar patterns were observed at the 3% cutoff and across all comparisons in Library 2, where effect sizes were consistently small (*R²* < 0.025).

At the genus and species level, no significant differences in the microbiome community structure were detected among the three filtering conditions under 0.5%, 1%, and 3% cutoffs for both libraries (*p* > 0.05) (Fig. 4C-F, Table S10). Effect sizes were generally small across all comparisons (*R²* < 0.03), indicating that filtering thresholds had minimal influence on overall community structure at finer taxonomic resolutions.

Table S1 Nanopore sequencing analysis comparison in the EPI2ME Agent by adjusting different parameters.

|  | Group ID | *Min_qscore* | *Min_coverage* | *Min_identity* | Average Alignment accuracy | Classification  success | Average quality | Workflow success | No. of Phyla |
| --- | --- | --- | --- | --- | --- | --- | --- | --- | --- |
| Library 1 | T1 | 9.00 | 60.00 | 90.00 | 92.00 | 41.00 | 10.22 | 47.10 | 13 |
|  | T2 | 9.00 | 60.00 | 95.00 | 96.00 | 1.00 | 10.22 | 47.10 | 6 |
|  | T3 | 9.00 | 80.00 | 90.00 | 92.00 | 41.00 | 10.22 | 47.10 | 13 |
|  | T4 | 8.00 | 60.00 | 90.00 | 92.00 | 47.00 | 10.22 | 53.10 | 13 |
|  | T5 | 8.00 | 80.00 | 95.00 | 96.00 | 1.00 | 10.22 | 53.10 | 6 |
|  | T6 | 7.00 | 60.00 | 90.00 | 88.00 | 98.00 | 10.24 | 53.80 | 22 |
|  | T7 | 7.00 | 30.00 | 95.00 | 96.00 | 1.00 | 10.23 | 53.50 | 6 |
|  | T8 | 8.00 | 30.00 | 95.00 | 96.00 | 1.00 | 10.22 | 53.00 | 6 |
|  | T9 | 9.00 | 30.00 | 95.00 | 95.47 | 1.00 | 10.22 | 47.00 | 6 |
|  | T10 | 8.00 | 30.00 | 77.00 | 86.11 | 99.00 | 10.40 | 70.20 | 23 |
|  | T11 | 9.00 | 30.00 | 77.00 | 86.78 | 100.00 | 10.39 | 61.90 | 23 |
|  | T0 | 7.00 | 30.00 | 77.00 | 86.14 | 99.00 | 10.37 | 65.30 | 23 |
| Library 2 | T1 | 9.00 | 60.00 | 90.00 | 92.00 | 61.00 | 10.60 | 76.50 | 12 |
|  | T2 | 9.00 | 60.00 | 95.00 | 95.00 | 3.00 | 10.60 | 76.70 | 8 |
|  | T3 | 9.00 | 80.00 | 90.00 | 91.42 | 60.00 | 10.60 | 76.30 | 12 |
|  | T4 | 8.00 | 60.00 | 90.00 | 92.00 | 55.00 | 10.60 | 84.10 | 12 |
|  | T5 | 8.00 | 80.00 | 95.00 | 95.42 | 2.00 | 10.60 | 84.40 | 8 |
|  | T6 | 7.00 | 60.00 | 90.00 | 92.00 | 53.00 | 10.59 | 87.40 | 12 |
|  | T7 | 7.00 | 30.00 | 95.00 | 95.43 | 2.00 | 10.60 | 88.00 | 8 |
|  | T8 | 8.00 | 30.00 | 95.00 | 95.44 | 2.00 | 10.60 | 84.60 | 8 |
|  | T9 | 9.00 | 30.00 | 95.00 | 95.43 | 3.00 | 10.60 | 76.60 | 7 |
|  | T10 | 8.00 | 30.00 | 77.00 | 90.00 | 100.00 | 10.59 | 84.00 | 23 |
|  | T11 | 9.00 | 30.00 | 77.00 | 90.00 | 100.00 | 10.59 | 76.70 | 23 |
|  | T0 | 7.00 | 30.00 | 77.00 | 85.41 | 99.00 | 10.59 | 87.50 | 24 |

Table S2 Summary of linear regression models for estimating the relationship between x and y.

Table S3 Summary of generalized linear mixed effects models for estimating the effects of *Min qscore*, *Min identity* and *Min coverage* on taxa characterization Alignment accuracy (Alignment accuracy) and classified proportion (Classification success).

|  | Dependent variable | Fixed effects | Estimate | Std. Error | *t* | *d.f.* | *p* | *Pseudo R² (Model)* | *Pseudo R² (Fixed effects)* | *Pseudo R² (Random effects)* |
| --- | --- | --- | --- | --- | --- | --- | --- | --- | --- | --- |
| Library 1 | Alignment accuracy | (Intercept) | 48.62 | 1.78 | 27.25 | 186.84 | 0.00 | 0.79 | 0.77 | 0.02 |
|  |  | ***Min_qscore*** | **0.87** | **0.14** | **6.05** | **184.00** | **0.00** |  |  |  |
|  |  | ***Min_identity*** | **0.44** | **0.02** | **25.84** | **184.00** | **0.00** |  |  |  |
|  |  | ***Min_coverage*** | **-0.05** | **0.01** | **-7.27** | **184.00** | **0.00** |  |  |  |
|  | Classification success | (Intercept) | 600.09 | 19.21 | 31.24 | 200.00 | 0.00 | 0.84 | 0.84 | 0.E+00 |
|  |  | ***Min_qscore*** | **-9.99** | **1.55** | **-6.46** | **200.00** | **0.00** |  |  |  |
|  |  | ***Min_identity*** | **-5.59** | **0.18** | **-30.64** | **200.00** | **0.00** |  |  |  |
|  |  | ***Min_coverage*** | **0.45** | **0.07** | **6.58** | **200.00** | **0.00** |  |  |  |
| Library 2 | Alignment accuracy | (Intercept) | 68.78 | 1.27 | 54.36 | 263.72 | 0.00 | 0.70 | 0.69 | 0.01 |
|  |  | *Min_qscore* | -0.11 | 0.10 | -1.05 | 261.00 | 0.29 |  |  |  |
|  |  | ***Min_identity*** | **0.29** | **0.01** | **24.28** | **261.00** | **0.00** |  |  |  |
|  |  | ***Min_coverage*** | **-0.01** | **0.00** | **-2.75** | **261.00** | **0.01** |  |  |  |
|  | Classification  success | (Intercept) | 474.05 | 20.57 | 23.05 | 284.00 | 0.00 | 0.72 | 0.72 | 0.E+00 |
|  |  | *Min_qscore* | 1.23 | 1.66 | 0.74 | 284.00 | 0.46 |  |  |  |
|  |  | ***Min_identity*** | **-5.10** | **0.20** | **-26.14** | **284.00** | **0.00** |  |  |  |
|  |  | ***Min_coverage*** | **0.20** | **0.07** | **2.75** | **284.00** | **0.01** |  |  |  |

Table S4 Statistics for the effect of *Min qscore* using Kruskal-Wallis rank sum test.

|  | Category | Group | ***Min qscore*** | *Min coverage* | *Min identity* | *χ^2^* | *d.f.* | *p* |
| --- | --- | --- | --- | --- | --- | --- | --- | --- |
| Library 1 | Alignment accuracy | T0, T10, T11 | 7,8,9 | 30 | 77 | 5.34 | 2 | 0.07 |
|  | Classification success | T0, T10, T11 | 7,8,9 | 30 | 77 | 2.40 | 2 | 0.30 |
|  | Phyla_0 | T0, T10, T11 | 7,8,9 | 30 | 77 | 0.05 | 2 | 0.97 |
|  | Phyla_0.1 | T0, T10, T11 | 7,8,9 | 30 | 77 | 0.41 | 2 | 0.82 |
|  | Phyla_0.5 | T0, T10, T11 | 7,8,9 | 30 | 77 | 0.51 | 2 | 0.77 |
|  | Phyla_1 | T0, T10, T11 | 7,8,9 | 30 | 77 | 0.34 | 2 | 0.84 |
|  | Phyla_3 | T0, T10, T11 | 7,8,9 | 30 | 77 | 0.06 | 2 | 0.97 |
| Library 2 | Alignment accuracy | T0, T10, T11 | 7,8,9 | 30 | 77 | 1.69 | 2 | 0.43 |
|  | Classification success | T0, T10, T11 | 7,8,9 | 30 | 77 | 4.02 | 2 | 0.13 |
|  | Phyla_0 | T0, T10, T11 | 7,8,9 | 30 | 77 | 19.24 | 2 | **0.00** |
|  | Phyla_0.1 | T0, T10, T11 | 7,8,9 | 30 | 77 | 13.39 | 2 | **0.00** |
|  | Phyla_0.5 | T0, T10, T11 | 7,8,9 | 30 | 77 | 8.97 | 2 | **0.01** |
|  | Phyla_1 | T0, T10, T11 | 7,8,9 | 30 | 77 | 5.33 | 2 | 0.07 |
|  | Phyla_3 | T0, T10, T11 | 7,8,9 | 30 | 77 | 1.88 | 2 | 0.39 |

Table S5 Statistics for the effect of *Min qscore* using Dunn’s pairwise tests.

|  |  | Group ID | Group | ***Min qscore*** | *Min coverage* | *Min identity* | *Z* | *P.unadj* | *P.adj* |
| --- | --- | --- | --- | --- | --- | --- | --- | --- | --- |
| Library 1 | Alignment accuracy | Group 1 | T0,T10 | 7,8 | 30 | 77 | 0.07 | 0.94 | 1.00 |
|  |  | Group 2 | T0,T11 | 7,9 | 30 | 77 | -1.96 | 0.05 | 0.15 |
|  |  | Group 3 | T10,T11 | 8,9 | 30 | 77 | -2.04 | 0.04 | 0.13 |
|  | Classification success | Group 1 | T0,T10 | 7,8 | 30 | 77 | 0.08 | 0.94 | 1.00 |
|  |  | Group 2 | T0,T11 | 7,9 | 30 | 77 | -1.30 | 0.19 | 0.58 |
|  |  | Group 3 | T10,T11 | 8,9 | 30 | 77 | -1.38 | 0.17 | 0.50 |
|  | Phyla_0 | Group 1 | T0,T10 | 7,8 | 30 | 77 | -0.06 | 0.95 | 1.00 |
|  |  | Group 2 | T0,T11 | 7,9 | 30 | 77 | 0.16 | 0.87 | 1.00 |
|  |  | Group 3 | T10,T11 | 8,9 | 30 | 77 | 0.22 | 0.83 | 1.00 |
|  | Phyla_0.1 | Group 1 | T0,T10 | 7,8 | 30 | 77 | 0.42 | 0.68 | 1.00 |
|  |  | Group 2 | T0,T11 | 7,9 | 30 | 77 | 0.63 | 0.53 | 1.00 |
|  |  | Group 3 | T10,T11 | 8,9 | 30 | 77 | 0.21 | 0.84 | 1.00 |
|  | Phyla_0.5 | Group 1 | T0,T10 | 7,8 | 30 | 77 | 0.71 | 0.48 | 1.00 |
|  |  | Group 2 | T0,T11 | 7,9 | 30 | 77 | 0.30 | 0.76 | 1.00 |
|  |  | Group 3 | T10,T11 | 8,9 | 30 | 77 | -0.41 | 0.68 | 1.00 |
|  | Phyla_1 | Group 1 | T0,T10 | 7,8 | 30 | 77 | 0.58 | 0.56 | 1.00 |
|  |  | Group 2 | T0,T11 | 7,9 | 30 | 77 | 0.27 | 0.78 | 1.00 |
|  |  | Group 3 | T10,T11 | 8,9 | 30 | 77 | -0.31 | 0.76 | 1.00 |
|  | Phyla_3 | Group 1 | T0,T10 | 7,8 | 30 | 77 | 0.00 | 1.00 | 1.00 |
|  |  | Group 2 | T0,T11 | 7,9 | 30 | 77 | -0.22 | 0.83 | 1.00 |
|  |  | Group 3 | T10,T11 | 8,9 | 30 | 77 | -0.22 | 0.83 | 1.00 |
| Library 2 | Alignment accuracy | Group 1 | T0,T10 | 7,8 | 30 | 77 | 1.12 | 0.26 | 0.78 |
|  |  | Group 2 | T0,T11 | 7,9 | 30 | 77 | 1.12 | 0.26 | 0.78 |
|  |  | Group 3 | T10,T11 | 8,9 | 30 | 77 | 0.00 | 1.00 | 1.00 |
|  | Classification success | Group 1 | T0,T10 | 7,8 | 30 | 77 | 1.70 | 0.09 | 0.27 |
|  |  | Group 2 | T0,T11 | 7,9 | 30 | 77 | 1.77 | 0.08 | 0.23 |
|  |  | Group 3 | T10,T11 | 8,9 | 30 | 77 | 0.08 | 0.94 | 1.00 |
|  | Phyla_0 | Group 1 | T0,T10 | 7,8 | 30 | 77 | -3.97 | 0.00 | **0.00** |
|  |  | Group 2 | T0,T11 | 7,9 | 30 | 77 | -3.60 | 0.00 | **0.00** |
|  |  | Group 3 | T10,T11 | 8,9 | 30 | 77 | 0.37 | 0.71 | 1.00 |
|  | Phyla_0.1 | Group 1 | T0,T10 | 7,8 | 30 | 77 | -3.12 | 0.00 | **0.01** |
|  |  | Group 2 | T0,T11 | 7,9 | 30 | 77 | -3.21 | 0.00 | **0.00** |
|  |  | Group 3 | T10,T11 | 8,9 | 30 | 77 | -0.09 | 0.93 | 1.00 |
|  | Phyla_0.5 | Group 1 | T0,T10 | 7,8 | 30 | 77 | -2.59 | 0.01 | **0.03** |
|  |  | Group 2 | T0,T11 | 7,9 | 30 | 77 | -2.59 | 0.01 | **0.03** |
|  |  | Group 3 | T10,T11 | 8,9 | 30 | 77 | 0.00 | 1.00 | 1.00 |
|  | Phyla_1 | Group 1 | T0,T10 | 7,8 | 30 | 77 | -2.05 | 0.04 | 0.12 |
|  |  | Group 2 | T0,T11 | 7,9 | 30 | 77 | -1.95 | 0.05 | 0.15 |
|  |  | Group 3 | T10,T11 | 8,9 | 30 | 77 | 0.10 | 0.92 | 1.00 |
|  | Phyla_3 | Group 1 | T0,T10 | 7,8 | 30 | 77 | -1.10 | 0.27 | 0.82 |
|  |  | Group 2 | T0,T11 | 7,9 | 30 | 77 | -1.26 | 0.21 | 0.62 |
|  |  | Group 3 | T10,T11 | 8,9 | 30 | 77 | -0.17 | 0.87 | 1.00 |

Table S6 Statistics for the effect of *Min coverage* using Wilcoxon signed rank tests.

|  |  | Group ID | Group | *Min qscore* | *Min coverage* | *Min identity* | *V* | *p* |
| --- | --- | --- | --- | --- | --- | --- | --- | --- |
| Library 1 | Alignment accuracy | Group 1 | T1, T3 | 9 | 60, 80 | 90 | 0 | NA |
|  |  | Group 2 | T5, T8 | 8 | 30, 80 | 95 | 0 | 0.15 |
|  | Classification success | Group 1 | T1, T3 | 9 | 60, 80 | 90 | 91 | **0.01** |
|  |  | Group 2 | T5, T8 | 8 | 30, 80 | 95 | 0 | NA |
|  | Phyla_0 | Group 1 | T1, T3 | 9 | 60, 80 | 90 | 153 | **0.00** |
|  |  | Group 2 | T5, T8 | 8 | 30, 80 | 95 | 0 | 1.00 |
|  | Phyla_0.1 | Group 1 | T1, T3 | 9 | 60, 80 | 90 | 120 | **0.00** |
|  |  | Group 2 | T5, T8 | 8 | 30, 80 | 95 | 0 | 0.35 |
|  | Phyla_0.5 | Group 1 | T1, T3 | 9 | 60, 80 | 90 | 136 | **0.00** |
|  |  | Group 2 | T5, T8 | 8 | 30, 80 | 95 | 3 | 0.35 |
|  | Phyla_1 | Group 1 | T1, T3 | 9 | 60, 80 | 90 | 75 | **0.00** |
|  |  | Group 2 | T5, T8 | 8 | 30, 80 | 95 | 0 | NA |
|  | Phyla_3 | Group 1 | T1, T3 | 9 | 60, 80 | 90 | 36 | **0.01** |
|  |  | Group 2 | T5, T8 | 8 | 30, 80 | 95 | 0 | NA |
| Library 2 | Alignment accuracy | Group 1 | T1, T3 | 9 | 60, 80 | 90 | 0 | **0.00** |
|  |  | Group 2 | T5, T8 | 8 | 30, 80 | 95 | 0 | **0.00** |
|  | Classification success | Group 1 | T1, T3 | 9 | 60, 80 | 90 | 300 | **0.00** |
|  |  | Group 2 | T5, T8 | 8 | 30, 80 | 95 | 300 | **0.00** |
|  | Phyla_0 | Group 1 | T1, T3 | 9 | 60, 80 | 90 | 1 | 1.00 |
|  |  | Group 2 | T5, T8 | 8 | 30, 80 | 95 | 0 | 1.00 |
|  | Phyla_0.1 | Group 1 | T1, T3 | 9 | 60, 80 | 90 | 1 | 1.00 |
|  |  | Group 2 | T5, T8 | 8 | 30, 80 | 95 | 0 | NA |
|  | Phyla_0.5 | Group 1 | T1, T3 | 9 | 60, 80 | 90 | 0 | 1.00 |
|  |  | Group 2 | T5, T8 | 8 | 30, 80 | 95 | 1 | 1.00 |
|  | Phyla_1 | Group 1 | T1, T3 | 9 | 60, 80 | 90 | 0 | 1.00 |
|  |  | Group 2 | T5, T8 | 8 | 30, 80 | 95 | 1.5 | 1.00 |
|  | Phyla_3 | Group 1 | T1, T3 | 9 | 60, 80 | 90 | 0 | NA |
|  |  | Group 2 | T5, T8 | 8 | 30, 80 | 95 | 0 | NA |

Table S7 Statistics for the effect of *Min identity* using Wilcoxon signed rank tests.

|  |  | Group ID | Group | *Min qscore* | *Min coverage* | ***Min identity*** | *V* | *p* |
| --- | --- | --- | --- | --- | --- | --- | --- | --- |
| Library 1 | Alignment accuracy | Group 1 | T1, T2 | 9 | 60 | 90, 95 | 0 | **0.00** |
|  |  | Group 2 | T0, T7 | 7 | 30 | 95, 77 | 0 | **0.00** |
|  | Classification success | Group 1 | T1, T2 | 9 | 60 | 90, 95 | 153 | **0.00** |
|  |  | Group 2 | T0, T7 | 7 | 30 | 95, 77 | 153 | **0.00** |
|  | Phyla_0 | Group 1 | T1, T2 | 9 | 60 | 90, 95 | 153 | **0.00** |
|  |  | Group 2 | T0, T7 | 7 | 30 | 95, 77 | 153 | **0.00** |
|  | Phyla_0.1 | Group 1 | T1, T2 | 9 | 60 | 90, 95 | 120 | **0.00** |
|  |  | Group 2 | T0, T7 | 7 | 30 | 95, 77 | 120 | **0.00** |
|  | Phyla_0.5 | Group 1 | T1, T2 | 9 | 60 | 90, 95 | 153 | **0.00** |
|  |  | Group 2 | T0, T7 | 7 | 30 | 95, 77 | 153 | **0.00** |
|  | Phyla_1 | Group 1 | T1, T2 | 9 | 60 | 90, 95 | 102.5 | **0.00** |
|  |  | Group 2 | T0, T7 | 7 | 30 | 95, 77 | 102.5 | **0.00** |
|  | Phyla_3 | Group 1 | T1, T2 | 9 | 60 | 90, 95 | 45 | **0.01** |
|  |  | Group 2 | T0, T7 | 7 | 30 | 95, 77 | 62 | **0.01** |
| Library 2 | Alignment accuracy | Group 1 | T1, T2 | 9 | 60 | 90, 95 | 0 | **0.00** |
|  |  | Group 2 | T0, T7 | 7 | 30 | 95, 77 | 0 | **0.00** |
|  | Classification success | Group 1 | T1, T2 | 9 | 60 | 90, 95 | 300 | **0.00** |
|  |  | Group 2 | T0, T7 | 7 | 30 | 95, 77 | 300 | **0.00** |
|  | Phyla_0 | Group 1 | T1, T2 | 9 | 60 | 90, 95 | 300 | **0.00** |
|  |  | Group 2 | T0, T7 | 7 | 30 | 95, 77 | 300 | **0.00** |
|  | Phyla_0.1 | Group 1 | T1, T2 | 9 | 60 | 90, 95 | 111 | **0.00** |
|  |  | Group 2 | T0, T7 | 7 | 30 | 95, 77 | 97 | **0.00** |
|  | Phyla_0.5 | Group 1 | T1, T2 | 9 | 60 | 90, 95 | 51 | **0.01** |
|  |  | Group 2 | T0, T7 | 7 | 30 | 95, 77 | 36 | **0.01** |
|  | Phyla_1 | Group 1 | T1, T2 | 9 | 60 | 90, 95 | 37 | 0.08 |
|  |  | Group 2 | T0, T7 | 7 | 30 | 95, 77 | 29 | 0.12 |
|  | Phyla_3 | Group 1 | T1, T2 | 9 | 60 | 90, 95 | 18 | 0.12 |
|  |  | Group 2 | T0, T7 | 7 | 30 | 95, 77 | 17.5 | 0.13 |

Table S8 Comparison among all 12 treatments using Kruskal-Wallis rank sum test

|  |  | Kruskal-Wallis chi-squared | *d.f.* | *p* |
| --- | --- | --- | --- | --- |
| Library 1 | Alignment accuracy | 187.80 | 11 | < 2.2e-16 |
|  | Classification success | 185.60 | 11 | < 2.2e-16 |
|  | Phyla_0 | 158.80 | 11 | 2.20E-16 |
|  | Phyla_0.1 | 109.88 | 11 | < 2.2e-16 |
|  | Phyla_0.5 | 115.49 | 11 | < 2.2e-16 |
|  | Phyla_1 | 67.21 | 11 | 4.14E-10 |
|  | Phyla_3 | 43.56 | 11 | 9.E-06 |
| Library 2 | Alignment accuracy | 254.08 | 11 | < 2.2e-16 |
|  | Classification success | 246.93 | 11 | < 2.2e-16 |
|  | Phyla_0 | 155.61 | 11 | < 2.2e-16 |
|  | Phyla_0.1 | 61.29 | 11 | 5.34E-09 |
|  | Phyla_0.5 | 35.03 | 11 | 2.E-04 |
|  | Phyla_1 | 22.08 | 11 | 0.02 |
|  | Phyla_3 | 7.55 | 11 | 0.75 |

Phyla_0: Cutoff = 0, no cutoff and all phyla detected were included. Phyla_0.1, Cutoff=0.1%, the number of detected phyla at 0.1% cutoff. Phyla_0.5, Cutoff = 0.5%, the number of detected phyla at 0.5% cutoff. Phyla_1, Cutoff = 1%, the number of detected phyla at 1% cutoff. Phyla_3, Cutoff = 3%, the number of detected phyla at 3% cutoff.

Table S9 Statistics for alpha diversity using Dunn's test with p-values adjusted by the Bonferroni method.

| Library | Taxon_level | Category | cutoff | Group | *d.f.* | χ2 | *p* |
| --- | --- | --- | --- | --- | --- | --- | --- |
| **1** | **phylum** | **Richness** | **0.5** | **T0,T1** | **2.61** | **0.01** | **0.03** |
| **1** | **phylum** | **Richness** | **0.5** | **T0,T2** | **5.27** | **0.00** | **0.00** |
| **1** | **phylum** | **Richness** | **0.5** | **T1,T2** | **2.66** | **0.01** | **0.02** |
| 1 | phylum | Richness | 1 | T0,T1 | 2.15 | 0.03 | 0.09 |
| 1 | phylum | Richness | 1 | T0,T2 | 3.86 | 0.00 | 0.00 |
| 1 | phylum | Richness | 1 | T1,T2 | 1.70 | 0.09 | 0.27 |
| 1 | phylum | Richness | 3 | T0,T1 | 1.56 | 0.12 | 0.36 |
| **1** | **phylum** | **Richness** | **3** | **T0,T2** | **2.83** | **0.00** | **0.01** |
| 1 | phylum | Richness | 3 | T1,T2 | 1.27 | 0.20 | 0.61 |
| **1** | **phylum** | **Shannon** | **0.5** | **T0,T1** | **3.03** | **0.00** | **0.01** |
| **1** | **phylum** | **Shannon** | **0.5** | **T0,T2** | **3.71** | **0.00** | **0.00** |
| 1 | phylum | Shannon | 0.5 | T1,T2 | 0.68 | 0.50 | 1.00 |
| **1** | **phylum** | **Shannon** | **1** | **T0,T1** | **2.81** | **0.00** | **0.01** |
| **1** | **phylum** | **Shannon** | **1** | **T0,T2** | **3.24** | **0.00** | **0.00** |
| 1 | phylum | Shannon | 1 | T1,T2 | 0.43 | 0.67 | 1.00 |
| **1** | **phylum** | **Shannon** | **3** | **T0,T1** | **2.81** | **0.00** | **0.01** |
| **1** | **phylum** | **Shannon** | **3** | **T0,T2** | **3.24** | **0.00** | **0.00** |
| 1 | phylum | Shannon | 3 | T1,T2 | 0.43 | 0.67 | 1.00 |
| 1 | phylum | Simpson | 0.5 | T0,T1 | 1.98 | 0.05 | 0.14 |
| 1 | phylum | Simpson | 0.5 | T0,T2 | 1.79 | 0.07 | 0.22 |
| 1 | phylum | Simpson | 0.5 | T1,T2 | -0.20 | 0.84 | 1.00 |
| 1 | phylum | Simpson | 1 | T0,T1 | 1.87 | 0.06 | 0.18 |
| 1 | phylum | Simpson | 1 | T0,T2 | 1.70 | 0.09 | 0.27 |
| 1 | phylum | Simpson | 1 | T1,T2 | -0.17 | 0.86 | 1.00 |
| 1 | phylum | Simpson | 3 | T0,T1 | 1.67 | 0.09 | 0.28 |
| 1 | phylum | Simpson | 3 | T0,T2 | 1.41 | 0.16 | 0.48 |
| 1 | phylum | Simpson | 3 | T1,T2 | -0.27 | 0.79 | 1.00 |
| 1 | phylum | Pielou's evenness | 0.5 | T0,T1 | 1.67 | 0.09 | 0.28 |
| 1 | phylum | Pielou's evenness | 0.5 | T0,T2 | 1.41 | 0.16 | 0.48 |
| 1 | phylum | Pielou's evenness | 0.5 | T1,T2 | -0.27 | 0.79 | 1.00 |
| 1 | phylum | Pielou's evenness | 1 | T0,T1 | 0.83 | 0.41 | 1.00 |
| 1 | phylum | Pielou's evenness | 1 | T0,T2 | -0.28 | 0.78 | 1.00 |
| 1 | phylum | Pielou's evenness | 1 | T1,T2 | -1.11 | 0.27 | 0.80 |
| 1 | phylum | Pielou's evenness | 3 | T0,T1 | 0.88 | 0.38 | 1.00 |
| 1 | phylum | Pielou's evenness | 3 | T0,T2 | -0.50 | 0.62 | 1.00 |
| 1 | phylum | Pielou's evenness | 3 | T1,T2 | -1.37 | 0.17 | 0.51 |
| 1 | Genus | Richness | 0.5 | T0,T1 | -0.88 | 0.38 | 1.00 |
| 1 | Genus | Richness | 0.5 | T0,T2 | -0.84 | 0.40 | 1.00 |
| 1 | Genus | Richness | 0.5 | T1,T2 | 0.04 | 0.97 | 1.00 |
| 1 | Genus | Richness | 1 | T0,T1 | -0.88 | 0.38 | 1.00 |
| 1 | Genus | Richness | 1 | T0,T2 | -0.84 | 0.40 | 1.00 |
| 1 | Genus | Richness | 1 | T1,T2 | 0.04 | 0.97 | 1.00 |
| 1 | Genus | Richness | 3 | T0,T1 | -2.18 | 0.03 | 0.09 |
| **1** | **Genus** | **Richness** | **3** | **T0,T2** | **-2.47** | **0.01** | **0.04** |
| 1 | Genus | Richness | 3 | T1,T2 | -0.29 | 0.77 | 1.00 |
| 1 | Genus | Shannon | 0.5 | T0,T1 | -0.18 | 0.85 | 1.00 |
| 1 | Genus | Shannon | 0.5 | T0,T2 | 0.88 | 0.38 | 1.00 |
| 1 | Genus | Shannon | 0.5 | T1,T2 | 1.06 | 0.29 | 0.87 |
| 1 | Genus | Shannon | 1 | T0,T1 | -0.18 | 0.85 | 1.00 |
| 1 | Genus | Shannon | 1 | T0,T2 | 0.88 | 0.38 | 1.00 |
| 1 | Genus | Shannon | 1 | T1,T2 | 1.06 | 0.29 | 0.87 |
| 1 | Genus | Shannon | 3 | T0,T1 | -0.18 | 0.85 | 1.00 |
| 1 | Genus | Shannon | 3 | T0,T2 | 0.88 | 0.38 | 1.00 |
| 1 | Genus | Shannon | 3 | T1,T2 | 1.06 | 0.29 | 0.87 |
| 1 | Genus | Simpson | 0.5 | T0,T1 | 0.02 | 0.98 | 1.00 |
| 1 | Genus | Simpson | 0.5 | T0,T2 | 1.08 | 0.28 | 0.83 |
| 1 | Genus | Simpson | 0.5 | T1,T2 | 1.06 | 0.29 | 0.87 |
| 1 | Genus | Simpson | 1 | T0,T1 | -0.47 | 0.64 | 1.00 |
| 1 | Genus | Simpson | 1 | T0,T2 | 0.58 | 0.56 | 1.00 |
| 1 | Genus | Simpson | 1 | T1,T2 | 1.05 | 0.29 | 0.88 |
| 1 | Genus | Simpson | 3 | T0,T1 | -1.67 | 0.09 | 0.28 |
| 1 | Genus | Simpson | 3 | T0,T2 | -1.06 | 0.29 | 0.87 |
| 1 | Genus | Simpson | 3 | T1,T2 | 0.61 | 0.54 | 1.00 |
| 1 | Genus | Pielou's evenness | 0.5 | T0,T1 | -1.67 | 0.09 | 0.28 |
| 1 | Genus | Pielou's evenness | 0.5 | T0,T2 | -1.06 | 0.29 | 0.87 |
| 1 | Genus | Pielou's evenness | 0.5 | T1,T2 | 0.61 | 0.54 | 1.00 |
| 1 | Genus | Pielou's evenness | 1 | T0,T1 | 0.88 | 0.38 | 1.00 |
| 1 | Genus | Pielou's evenness | 1 | T0,T2 | 2.13 | 0.03 | 0.10 |
| 1 | Genus | Pielou's evenness | 1 | T1,T2 | 1.26 | 0.21 | 0.63 |
| 1 | Genus | Pielou's evenness | 3 | T0,T1 | 1.38 | 0.17 | 0.50 |
| 1 | Genus | Pielou's evenness | 3 | T0,T2 | 1.83 | 0.07 | 0.20 |
| 1 | Genus | Pielou's evenness | 3 | T1,T2 | 0.45 | 0.65 | 1.00 |
| 1 | Species | Richness | 0.5 | T0,T1 | -1.57 | 0.12 | 0.35 |
| 1 | Species | Richness | 0.5 | T0,T2 | -2.09 | 0.04 | 0.11 |
| 1 | Species | Richness | 0.5 | T1,T2 | -0.53 | 0.60 | 1.00 |
| 1 | Species | Richness | 1 | T0,T1 | -2.34 | 0.02 | 0.06 |
| **1** | **Species** | **Richness** | **1** | **T0,T2** | **-2.65** | **0.01** | **0.02** |
| 1 | Species | Richness | 1 | T1,T2 | -0.31 | 0.76 | 1.00 |
| 1 | Species | Richness | 3 | T0,T1 | -2.30 | 0.02 | 0.06 |
| **1** | **Species** | **Richness** | **3** | **T0,T2** | **-3.68** | **0.00** | **0.00** |
| 1 | Species | Richness | 3 | T1,T2 | -1.38 | 0.17 | 0.50 |
| 1 | Species | Shannon | 0.5 | T0,T1 | -0.85 | 0.39 | 1.00 |
| 1 | Species | Shannon | 0.5 | T0,T2 | -0.88 | 0.38 | 1.00 |
| 1 | Species | Shannon | 0.5 | T1,T2 | -0.02 | 0.98 | 1.00 |
| 1 | Species | Shannon | 1 | T0,T1 | -1.57 | 0.12 | 0.35 |
| 1 | Species | Shannon | 1 | T0,T2 | -1.51 | 0.13 | 0.39 |
| 1 | Species | Shannon | 1 | T1,T2 | 0.06 | 0.95 | 1.00 |
| **1** | **Species** | **Shannon** | **3** | **T0,T1** | **-2.43** | **0.01** | **0.04** |
| **1** | **Species** | **Shannon** | **3** | **T0,T2** | **-3.10** | **0.00** | **0.01** |
| 1 | Species | Shannon | 3 | T1,T2 | -0.67 | 0.50 | 1.00 |
| 1 | Species | Simpson | 0.5 | T0,T1 | -0.62 | 0.53 | 1.00 |
| 1 | Species | Simpson | 0.5 | T0,T2 | -0.07 | 0.94 | 1.00 |
| 1 | Species | Simpson | 0.5 | T1,T2 | 0.55 | 0.58 | 1.00 |
| 1 | Species | Simpson | 1 | T0,T1 | -1.26 | 0.21 | 0.63 |
| 1 | Species | Simpson | 1 | T0,T2 | -0.47 | 0.64 | 1.00 |
| 1 | Species | Simpson | 1 | T1,T2 | 0.78 | 0.43 | 1.00 |
| 1 | Species | Simpson | 3 | T0,T1 | -2.26 | 0.02 | 0.07 |
| **1** | **Species** | **Simpson** | **3** | **T0,T2** | **-2.62** | **0.01** | **0.03** |
| 1 | Species | Simpson | 3 | T1,T2 | -0.36 | 0.72 | 1.00 |
| 1 | Species | Pielou's evenness | 0.5 | T0,T1 | 0.74 | 0.46 | 1.00 |
| 1 | Species | Pielou's evenness | 0.5 | T0,T2 | 1.82 | 0.07 | 0.21 |
| 1 | Species | Pielou's evenness | 0.5 | T1,T2 | 1.08 | 0.28 | 0.83 |
| 1 | Species | Pielou's evenness | 1 | T0,T1 | 0.85 | 0.39 | 1.00 |
| 1 | Species | Pielou's evenness | 1 | T0,T2 | 1.92 | 0.06 | 0.17 |
| 1 | Species | Pielou's evenness | 1 | T1,T2 | 1.06 | 0.29 | 0.87 |
| 1 | Species | Pielou's evenness | 3 | T0,T1 | -0.58 | 0.56 | 1.00 |
| 1 | Species | Pielou's evenness | 3 | T0,T2 | 0.58 | 0.56 | 1.00 |
| 1 | Species | Pielou's evenness | 3 | T1,T2 | 1.15 | 0.25 | 0.75 |
| 2 | phylum | Richness | 0.5 | T0,T1 | 2.39 | 0.02 | 0.05 |
| **2** | **phylum** | **Richness** | **0.5** | **T0,T2** | **3.60** | **0.00** | **0.00** |
| 2 | phylum | Richness | 0.5 | T1,T2 | 1.21 | 0.23 | 0.68 |
| 2 | phylum | Richness | 1 | T0,T1 | 1.77 | 0.08 | 0.23 |
| **2** | **phylum** | **Richness** | **1** | **T0,T2** | **2.89** | **0.00** | **0.01** |
| 2 | phylum | Richness | 1 | T1,T2 | 1.12 | 0.26 | 0.79 |
| 2 | phylum | Richness | 3 | T0,T1 | 1.77 | 0.08 | 0.23 |
| **2** | **phylum** | **Richness** | **3** | **T0,T2** | **2.89** | **0.00** | **0.01** |
| 2 | phylum | Richness | 3 | T1,T2 | 1.12 | 0.26 | 0.79 |
| 2 | phylum | Shannon | 0.5 | T0,T1 | 1.66 | 0.10 | 0.29 |
| **2** | **phylum** | **Shannon** | **0.5** | **T0,T2** | **2.36** | **0.02** | **0.05** |
| 2 | phylum | Shannon | 0.5 | T1,T2 | 0.70 | 0.48 | 1.00 |
| 2 | phylum | Shannon | 1 | T0,T1 | 1.45 | 0.15 | 0.44 |
| 2 | phylum | Shannon | 1 | T0,T2 | 2.20 | 0.03 | 0.08 |
| 2 | phylum | Shannon | 1 | T1,T2 | 0.75 | 0.46 | 1.00 |
| 2 | phylum | Shannon | 3 | T0,T1 | 1.14 | 0.25 | 0.76 |
| 2 | phylum | Shannon | 3 | T0,T2 | 1.61 | 0.11 | 0.32 |
| 2 | phylum | Shannon | 3 | T1,T2 | 0.47 | 0.64 | 1.00 |
| 2 | phylum | Simpson | 0.5 | T0,T1 | 1.36 | 0.17 | 0.52 |
| 2 | phylum | Simpson | 0.5 | T0,T2 | 1.95 | 0.05 | 0.15 |
| 2 | phylum | Simpson | 0.5 | T1,T2 | 0.59 | 0.55 | 1.00 |
| 2 | phylum | Simpson | 1 | T0,T1 | 1.29 | 0.20 | 0.59 |
| 2 | phylum | Simpson | 1 | T0,T2 | 1.82 | 0.07 | 0.21 |
| 2 | phylum | Simpson | 1 | T1,T2 | 0.53 | 0.60 | 1.00 |
| 2 | phylum | Simpson | 3 | T0,T1 | 1.02 | 0.31 | 0.92 |
| 2 | phylum | Simpson | 3 | T0,T2 | 1.35 | 0.18 | 0.53 |
| 2 | phylum | Simpson | 3 | T1,T2 | 0.33 | 0.74 | 1.00 |
| 2 | phylum | Pielou's evenness | 0.5 | T0,T1 | 0.60 | 0.55 | 1.00 |
| 2 | phylum | Pielou's evenness | 0.5 | T0,T2 | 1.06 | 0.29 | 0.87 |
| 2 | phylum | Pielou's evenness | 0.5 | T1,T2 | 0.46 | 0.65 | 1.00 |
| 2 | phylum | Pielou's evenness | 1 | T0,T1 | 0.53 | 0.59 | 1.00 |
| 2 | phylum | Pielou's evenness | 1 | T0,T2 | 0.96 | 0.34 | 1.00 |
| 2 | phylum | Pielou's evenness | 1 | T1,T2 | 0.43 | 0.67 | 1.00 |
| 2 | phylum | Pielou's evenness | 3 | T0,T1 | 0.64 | 0.52 | 1.00 |
| 2 | phylum | Pielou's evenness | 3 | T0,T2 | 0.72 | 0.47 | 1.00 |
| 2 | phylum | Pielou's evenness | 3 | T1,T2 | 0.07 | 0.94 | 1.00 |
| 2 | Genus | Richness | 0.5 | T0,T1 | -0.50 | 0.62 | 1.00 |
| 2 | Genus | Richness | 0.5 | T0,T2 | 0.14 | 0.89 | 1.00 |
| 2 | Genus | Richness | 0.5 | T1,T2 | 0.64 | 0.52 | 1.00 |
| 2 | Genus | Richness | 1 | T0,T1 | -0.32 | 0.75 | 1.00 |
| 2 | Genus | Richness | 1 | T0,T2 | -0.44 | 0.66 | 1.00 |
| 2 | Genus | Richness | 1 | T1,T2 | -0.12 | 0.90 | 1.00 |
| 2 | Genus | Richness | 3 | T0,T1 | -0.75 | 0.46 | 1.00 |
| 2 | Genus | Richness | 3 | T0,T2 | -1.41 | 0.16 | 0.48 |
| 2 | Genus | Richness | 3 | T1,T2 | -0.66 | 0.51 | 1.00 |
| 2 | Genus | Shannon | 0.5 | T0,T1 | -0.13 | 0.90 | 1.00 |
| 2 | Genus | Shannon | 0.5 | T0,T2 | 0.44 | 0.66 | 1.00 |
| 2 | Genus | Shannon | 0.5 | T1,T2 | 0.57 | 0.57 | 1.00 |
| 2 | Genus | Shannon | 1 | T0,T1 | -0.11 | 0.91 | 1.00 |
| 2 | Genus | Shannon | 1 | T0,T2 | 0.11 | 0.91 | 1.00 |
| 2 | Genus | Shannon | 1 | T1,T2 | 0.22 | 0.83 | 1.00 |
| 2 | Genus | Shannon | 3 | T0,T1 | -0.31 | 0.76 | 1.00 |
| 2 | Genus | Shannon | 3 | T0,T2 | -0.66 | 0.51 | 1.00 |
| 2 | Genus | Shannon | 3 | T1,T2 | -0.35 | 0.72 | 1.00 |
| 2 | Genus | Simpson | 0.5 | T0,T1 | 0.11 | 0.91 | 1.00 |
| 2 | Genus | Simpson | 0.5 | T0,T2 | 0.55 | 0.58 | 1.00 |
| 2 | Genus | Simpson | 0.5 | T1,T2 | 0.44 | 0.66 | 1.00 |
| 2 | Genus | Simpson | 1 | T0,T1 | 0.12 | 0.91 | 1.00 |
| 2 | Genus | Simpson | 1 | T0,T2 | 0.32 | 0.75 | 1.00 |
| 2 | Genus | Simpson | 1 | T1,T2 | 0.20 | 0.84 | 1.00 |
| 2 | Genus | Simpson | 3 | T0,T1 | -0.09 | 0.93 | 1.00 |
| 2 | Genus | Simpson | 3 | T0,T2 | -0.37 | 0.71 | 1.00 |
| 2 | Genus | Simpson | 3 | T1,T2 | -0.28 | 0.78 | 1.00 |
| 2 | Genus | Pielou's evenness | 0.5 | T0,T1 | 0.53 | 0.60 | 1.00 |
| 2 | Genus | Pielou's evenness | 0.5 | T0,T2 | 0.86 | 0.39 | 1.00 |
| 2 | Genus | Pielou's evenness | 0.5 | T1,T2 | 0.32 | 0.75 | 1.00 |
| 2 | Genus | Pielou's evenness | 1 | T0,T1 | 0.53 | 0.60 | 1.00 |
| 2 | Genus | Pielou's evenness | 1 | T0,T2 | 0.90 | 0.37 | 1.00 |
| 2 | Genus | Pielou's evenness | 1 | T1,T2 | 0.37 | 0.71 | 1.00 |
| 2 | Genus | Pielou's evenness | 3 | T0,T1 | 0.58 | 0.56 | 1.00 |
| 2 | Genus | Pielou's evenness | 3 | T0,T2 | 0.77 | 0.44 | 1.00 |
| 2 | Genus | Pielou's evenness | 3 | T1,T2 | 0.19 | 0.85 | 1.00 |
| 2 | Species | Richness | 0.5 | T0,T1 | -0.89 | 0.37 | 1.00 |
| 2 | Species | Richness | 0.5 | T0,T2 | -0.36 | 0.72 | 1.00 |
| 2 | Species | Richness | 0.5 | T1,T2 | 0.54 | 0.59 | 1.00 |
| 2 | Species | Richness | 1 | T0,T1 | -0.46 | 0.65 | 1.00 |
| 2 | Species | Richness | 1 | T0,T2 | -0.99 | 0.32 | 0.96 |
| 2 | Species | Richness | 1 | T1,T2 | -0.53 | 0.59 | 1.00 |
| 2 | Species | Richness | 3 | T0,T1 | -1.18 | 0.24 | 0.72 |
| **2** | **Species** | **Richness** | **3** | **T0,T2** | **-2.47** | **0.01** | **0.04** |
| 2 | Species | Richness | 3 | T1,T2 | -1.29 | 0.20 | 0.59 |
| 2 | Species | Shannon | 0.5 | T0,T1 | -2.30 | 0.02 | 0.06 |
| **2** | **Species** | **Shannon** | **0.5** | **T0,T2** | **-3.68** | **0.00** | **0.00** |
| 2 | Species | Shannon | 0.5 | T1,T2 | -1.38 | 0.17 | 0.50 |
| 2 | Species | Shannon | 1 | T0,T1 | -2.30 | 0.02 | 0.06 |
| **2** | **Species** | **Shannon** | **1** | **T0,T2** | **-3.68** | **0.00** | **0.00** |
| 2 | Species | Shannon | 1 | T1,T2 | -1.38 | 0.17 | 0.50 |
| 2 | Species | Shannon | 3 | T0,T1 | -0.57 | 0.57 | 1.00 |
| 2 | Species | Shannon | 3 | T0,T2 | -2.04 | 0.04 | 0.13 |
| 2 | Species | Shannon | 3 | T1,T2 | -1.46 | 0.14 | 0.43 |
| 2 | Species | Simpson | 0.5 | T0,T1 | -0.07 | 0.95 | 1.00 |
| 2 | Species | Simpson | 0.5 | T0,T2 | -0.28 | 0.78 | 1.00 |
| 2 | Species | Simpson | 0.5 | T1,T2 | -0.21 | 0.83 | 1.00 |
| 2 | Species | Simpson | 1 | T0,T1 | -0.07 | 0.95 | 1.00 |
| 2 | Species | Simpson | 1 | T0,T2 | -0.28 | 0.78 | 1.00 |
| 2 | Species | Simpson | 1 | T1,T2 | -0.21 | 0.83 | 1.00 |
| 2 | Species | Simpson | 3 | T0,T1 | -0.33 | 0.74 | 1.00 |
| 2 | Species | Simpson | 3 | T0,T2 | -1.66 | 0.10 | 0.29 |
| 2 | Species | Simpson | 3 | T1,T2 | -1.32 | 0.19 | 0.56 |
| 2 | Species | Pielou's evenness | 0.5 | T0,T1 | 0.35 | 0.73 | 1.00 |
| 2 | Species | Pielou's evenness | 0.5 | T0,T2 | 0.19 | 0.85 | 1.00 |
| 2 | Species | Pielou's evenness | 0.5 | T1,T2 | -0.17 | 0.87 | 1.00 |
| 2 | Species | Pielou's evenness | 1 | T0,T1 | 0.35 | 0.73 | 1.00 |
| 2 | Species | Pielou's evenness | 1 | T0,T2 | 0.27 | 0.79 | 1.00 |
| 2 | Species | Pielou's evenness | 1 | T1,T2 | -0.08 | 0.93 | 1.00 |
| 2 | Species | Pielou's evenness | 3 | T0,T1 | 0.08 | 0.94 | 1.00 |
| 2 | Species | Pielou's evenness | 3 | T0,T2 | -0.12 | 0.91 | 1.00 |
| 2 | Species | Pielou's evenness | 3 | T1,T2 | -0.19 | 0.85 | 1.00 |

Table S10 Statistics for microbiome structure components comparison by PERMANOVA.

| Library | Taxon level | Cutoff | Group | *Pseudo-F* | *R²* | *p* |
| --- | --- | --- | --- | --- | --- | --- |
| 1 | Phylum | 0.50% | T1 vs T2 | 1.12 | 0.03 | 1.00 |
| 1 | Phylum | 0.50% | T1 vs T0 | 3.13 | 0.09 | 1.00 |
| 1 | Phylum | 0.50% | T2 vs T0 | 3.01 | 0.09 | 1.00 |
| 1 | Phylum | 1% | T1 vs T2 | 1.05 | 0.03 | 1.00 |
| 1 | Phylum | 1% | T1 vs T0 | 2.94 | 0.08 | 1.00 |
| 1 | Phylum | 1% | T2 vs T0 | 2.71 | 0.08 | 1.00 |
| 1 | Phylum | 3% | T1 vs T2 | 0.98 | 0.03 | 1.00 |
| 1 | Phylum | 3% | T1 vs T0 | 2.51 | 0.07 | 1.00 |
| 1 | Phylum | 3% | T2 vs T0 | 2.28 | 0.07 | 1.00 |
| 1 | Genus | 0.50% | T1 vs T2 | 0.67 | 0.02 | 1.00 |
| 1 | Genus | 0.50% | T1 vs T0 | 0.08 | 0.00 | 1.00 |
| 1 | Genus | 0.50% | T2 vs T0 | 0.95 | 0.03 | 1.00 |
| 1 | Genus | 1% | T1 vs T2 | 0.68 | 0.02 | 1.00 |
| 1 | Genus | 1% | T1 vs T0 | 0.06 | 0.00 | 1.00 |
| 1 | Genus | 1% | T2 vs T0 | 0.92 | 0.03 | 1.00 |
| 1 | Genus | 3% | T1 vs T2 | 0.54 | 0.02 | 1.00 |
| 1 | Genus | 3% | T1 vs T0 | 0.14 | 0.00 | 1.00 |
| 1 | Genus | 3% | T2 vs T0 | 0.66 | 0.02 | 1.00 |
| 1 | Species | 0.50% | T1 vs T2 | 0.55 | 0.02 | 1.00 |
| 1 | Species | 0.50% | T1 vs T0 | 0.08 | 0.00 | 1.00 |
| 1 | Species | 0.50% | T2 vs T0 | 0.75 | 0.02 | 1.00 |
| 1 | Species | 1% | T1 vs T2 | 0.53 | 0.02 | 1.00 |
| 1 | Species | 1% | T1 vs T0 | 0.10 | 0.00 | 1.00 |
| 1 | Species | 1% | T2 vs T0 | 0.65 | 0.02 | 1.00 |
| 1 | Species | 3% | T1 vs T2 | 0.45 | 0.01 | 1.00 |
| 1 | Species | 3% | T1 vs T0 | 0.15 | 0.00 | 1.00 |
| 1 | Species | 3% | T2 vs T0 | 0.71 | 0.02 | 1.00 |
| 2 | Phylum | 0.50% | T1 vs T2 | 0.12 | 0.00 | 1.00 |
| 2 | Phylum | 0.50% | T1 vs T0 | 0.67 | 0.01 | 1.00 |
| 2 | Phylum | 0.50% | T2 vs T0 | 1.14 | 0.02 | 1.00 |
| 2 | Phylum | 1% | T1 vs T2 | 0.10 | 0.00 | 1.00 |
| 2 | Phylum | 1% | T1 vs T0 | 0.54 | 0.01 | 1.00 |
| 2 | Phylum | 1% | T2 vs T0 | 0.94 | 0.02 | 1.00 |
| 2 | Phylum | 3% | T1 vs T2 | 0.06 | 0.00 | 1.00 |
| 2 | Phylum | 3% | T1 vs T0 | 0.36 | 0.01 | 1.00 |
| 2 | Phylum | 3% | T2 vs T0 | 0.55 | 0.01 | 1.00 |
| 2 | Genus | 0.50% | T1 vs T2 | 0.51 | 0.01 | 1.00 |
| 2 | Genus | 0.50% | T1 vs T0 | 0.05 | 0.00 | 1.00 |
| 2 | Genus | 0.50% | T2 vs T0 | 0.70 | 0.01 | 1.00 |
| 2 | Genus | 1% | T1 vs T2 | 0.46 | 0.01 | 1.00 |
| 2 | Genus | 1% | T1 vs T0 | 0.03 | 0.00 | 1.00 |
| 2 | Genus | 1% | T2 vs T0 | 0.61 | 0.01 | 1.00 |
| 2 | Genus | 3% | T1 vs T2 | 0.33 | 0.01 | 1.00 |
| 2 | Genus | 3% | T1 vs T0 | 0.03 | 0.00 | 1.00 |
| 2 | Genus | 3% | T2 vs T0 | 0.39 | 0.01 | 1.00 |
| 2 | Species | 0.50% | T1 vs T2 | 0.40 | 0.01 | 1.00 |
| 2 | Species | 0.50% | T1 vs T0 | 0.05 | 0.00 | 1.00 |
| 2 | Species | 0.50% | T2 vs T0 | 0.49 | 0.01 | 1.00 |
| 2 | Species | 1% | T1 vs T2 | 0.32 | 0.01 | 1.00 |
| 2 | Species | 1% | T1 vs T0 | 0.04 | 0.00 | 1.00 |
| 2 | Species | 1% | T2 vs T0 | 0.42 | 0.01 | 1.00 |
| 2 | Species | 3% | T1 vs T2 | 0.33 | 0.01 | 1.00 |
| 2 | Species | 3% | T1 vs T0 | 0.06 | 0.00 | 1.00 |
| 2 | Species | 3% | T2 vs T0 | 0.46 | 0.01 | 1.00 |

Table S11 Statistics for relative abundance of taxa using Kruskal-Wallis rank sum test.

| Library | Taxon_  level | cutoff | Group | Taxon | *d.f.* | χ2 | *p* |
| --- | --- | --- | --- | --- | --- | --- | --- |
| 1 | Phylum | 3.00% | T0, T1, T2 | Proteobacteria | 2 | 2.19 | 0.34 |
| 1 | Phylum | 3.00% | T0, T1, T2 | Firmicutes | 2 | 0.45 | 0.80 |
| 1 | Phylum | 3.00% | T0, T1, T2 | Actinobacteria | 2 | 2.09 | 0.35 |
| **1** | **Phylum** | **3.00%** | **T0, T1, T2** | **Cyanobacteria** | **2** | **24.01** | **0.00** |
| 1 | Phylum | 3.00% | T0, T1, T2 | Bacteroidetes | 2 | 1.56 | 0.46 |
| 1 | Phylum | 3.00% | T0, T1, T2 | Acidobacteria | 2 | 2.08 | 0.35 |
| 1 | Genus | 3.00% | T0, T1, T2 | *Ralstonia* | 2 | 1.30 | 0.52 |
| 1 | Genus | 3.00% | T0, T1, T2 | *Rhodococcus* | 2 | 0.50 | 0.78 |
| 1 | Genus | 3.00% | T0, T1, T2 | *Paraburkholderia* | 2 | 0.07 | 0.97 |
| 1 | Genus | 3.00% | T0, T1, T2 | *Pseudomonas* | 2 | 1.24 | 0.54 |
| 1 | Genus | 3.00% | T0, T1, T2 | *Vagococcus* | 2 | 0.03 | 0.90 |
| 1 | Genus | 3.00% | T0, T1, T2 | *Massilia* | 2 | 2.56 | 0.28 |
| 1 | Species | 3.00% | T0, T1, T2 | *Ralstonia.pickettii* | 2 | 0.25 | 0.88 |
| 1 | Species | 3.00% | T0, T1, T2 | *Rhodococcus.qingshengii* | 2 | 1.93 | 0.38 |
| 1 | Species | 3.00% | T0, T1, T2 | *Stenotrophomonas.rhizophila* | 2 | 0.65 | 0.72 |
| 1 | Species | 3.00% | T0, T1, T2 | *Paraburkholderia.fungorum* | 2 | 0.10 | 0.95 |
| 2 | Phylum | 3.00% | T0, T1, T2 | Proteobacteria | 2 | 1.35 | 0.51 |
| 2 | Phylum | 3.00% | T0, T1, T2 | Firmicutes | 2 | 0.19 | 0.91 |
| 2 | Phylum | 3.00% | T0, T1, T2 | Actinobacteria | 2 | 0.57 | 0.75 |
| **2** | **Phylum** | **3.00%** | **T0, T1, T2** | **Cyanobacteria** | **2** | **17.67** | **0.00** |
| 2 | Phylum | 3.00% | T0, T1, T2 | Bacteroidetes | 2 | 4.51 | 0.10 |
| 2 | Phylum | 3.00% | T0, T1, T2 | Acidobacteria | 2 | 2.06 | 0.36 |
| 2 | Genus | 3.00% | T0, T1, T2 | *Ralstonia* | 2 | 0.77 | 0.68 |
| 2 | Genus | 3.00% | T0, T1, T2 | *Rhodococcus* | 2 | 0.00 | 1.00 |
| 2 | Genus | 3.00% | T0, T1, T2 | *Paraburkholderia* | 2 | 0.12 | 0.94 |
| 2 | Genus | 3.00% | T0, T1, T2 | *Pseudomonas* | 2 | 0.11 | 0.11 |
| 2 | Genus | 3.00% | T0, T1, T2 | *Vagococcus* | 2 | 0.99 | 0.61 |
| 2 | Genus | 3.00% | T0, T1, T2 | *Massilia* | 2 | 1.23 | 0.54 |
| 2 | Species | 3.00% | T0, T1, T2 | *Ralstonia.pickettii* | 2 | 0.43 | 0.81 |
| 2 | Species | 3.00% | T0, T1, T2 | *Rhodococcus.qingshengii* | 2 | 0.00 | 1.00 |
| 2 | Species | 3.00% | T0, T1, T2 | *Stenotrophomonas.rhizophila* | 2 | 0.61 | 0.74 |
| 2 | Species | 3.00% | T0, T1, T2 | *Paraburkholderia.fungorum* | 2 | 0.18 | 0.91 |

Table S12 Top 10 classified taxa as Cyanobacteria in S12 from Library 1, collected from the EPI2ME Agent.

Figure S1 Effects of filtering conditions (T0–T11) on the Alignment accuracy, Classification success, and phylum detection in Library 1 and 2. (A) Alignment accuracy under different filtering conditions. (B) Classification success under different filtering conditions. The number of phyla detected under different filtering methods (T0–T11) at different cutoffs in Library 1 and 2: (C) Cutoff = 0, no cutoff and all phyla detected were included. (D) Cutoff = 0.5%, the number of detected phyla at 0.5% cutoff. (E) Cutoff = 1%, the number of detected phyla at 1% cutoff. (F) Cutoff = 3%, the number of detected phyla at 3% cutoff.

Figure S2 Linear regression showing the negative correlation between Alignment accuracy and Classification success under 12 conditions. Regression statistics, *p* < 0.001; Library 1, *β* = -11.57, *F*_1,202_ = 1428.00, _adjusted_*R^2^* = 0.88; Library 2, *β* = -16.12, *F*_1,286_ = 2289.00, _adjusted_*R^2^* = 0.89.

Figure S3 Alignment accuracy for Cyanobacteria (left lane) and Proteobacteria (right lane) in Library 1 under T0 filtering condition. Samples, S12 and S13.

A

B

C

D

E

Figure S4 Species-level characterization for S12 from Library1 at different abundance cutoffs collected from the EPI2ME Agent. (A) no cutoff (0). (B) 0.1% cutoff. (C) 0.5% cutoff. (D) 1% cutoff. (E) 3% cutoff.
